# Identification of Distant Agouti-Like Sequences and Re-Evaluation of the Evolutionary History of the Agouti-Related Peptide (*AgRP*)

**DOI:** 10.1371/journal.pone.0040982

**Published:** 2012-07-16

**Authors:** Åke Västermark, Arunkumar Krishnan, Michael E. Houle, Robert Fredriksson, José Miguel Cerdá-Reverter, Helgi B. Schiöth

**Affiliations:** 1 Department of Neuroscience, Uppsala University, Uppsala, Sweden; 2 National Institute of Informatics, Research Organization of Information and Systems, Tokyo, Japan; 3 Instituto de Acuicultura de Torre de la Sal, Consejo Superior de Investigaciones Científicas, Castellón, Spain; Ecole Normale Supérieure de Lyon, France

## Abstract

The Agouti-like peptides including *AgRP*, *ASIP* and the teleost-specific A2 (*ASIP2* and *AgRP2*) peptides have potent and diverse functional roles in feeding, pigmentation and background adaptation mechanisms. There are contradictory theories about the evolution of the Agouti-like peptide family as well the nomenclature. Here we performed comprehensive mining and annotation of vertebrate Agouti-like sequences. We identified A2 sequences from salmon, trout, seabass, cod, cichlid, tilapia, gilt-headed sea bream, Antarctic toothfish, rainbow smelt, common carp, channel catfish and interestingly also in lobe-finned fish. Moreover, we surprisingly found eight novel homologues from the kingdom of arthropods and three from fungi, some sharing the characteristic C-x(6)-C-C motif which are present in the Agouti-like sequences, as well as approximate sequence length (130 amino acids), positioning of the motif sequence and sharing of exon-intron structures that are similar to the other Agouti-like peptides providing further support for the common origin of these sequences. Phylogenetic analysis shows that the *AgRP* sequences cluster basally in the tree, suggesting that these sequences split from a cluster containing both the *ASIP* and the A2 sequences. We also used a novel approach to determine the statistical evidence for synteny, a sinusoidal Hough transform pattern recognition technique. Our analysis shows that the teleost *AgRP2* resides in a chromosomal region that has synteny with Hsa 8, but we found no convincing synteny between the regions that A2, *AgRP* and *ASIP* reside in, which would support that the Agouti-like peptides were formed by whole genome tetraplodization events. Here we suggest that the Agouti-like peptide genes were formed through classical subsequent gene duplications where the *AgRP* is the most distantly related to the three other members of that group, first splitting from a common ancestor to *ASIP* and A2, and then later the A2 split from *ASIP* followed by a split resulting in *ASIP2* and *AgRP2*.

## Introduction

Agouti signaling peptide (*ASIP*) was discovered in 1993 [1] while the Agouti-related peptide (*AgRP*) was first identified in 1997 [2], [3]. The word *Agouti*, from the Guaraní language of South America, refers to rodents noted for prominent-banded pigment patterns in individual hair shafts. Made from three coding exons, these proteins are approximately 130 amino acids long, and contain a cysteine knot (receptor binding domain) in the third coding exon. The cysteine knot mediates the function of ASIP as an inverse agonist of melanocortin (MC) 1 and 4 receptors [4] while *AgRP* acts mainly at the MC3 and MC4 receptors [5]. The effect of *ASIP* on mammalian hair follicle melanocytes is an increased production of pheomelanin (yellow) and a decreased production of eumelanin (brown/black). It helps to establish the dorsal-ventral pigmentation in goldfish, by being mainly expressed in ventral skin, where it inhibits melanophore differentiation and/or proliferation but promotes iridophore differentiation and/or proliferation [6]. *AgRP* is one of the most potent appetite stimulants within the hypothalamus, and it plays an important role in mediating the effects of the peripheral body weight regulators ghrelin and leptin [7].

In 2003, we cloned the first *AgRP* sequences in fish [8], [9]. Then, in 2005, we searched for *ASIP* and *AgRP* sequences in fish and chicken and found a third category of Agouti-like proteins, which have a cysteine knot that has been shortened by one amino acid to give a C-x(6)-C-x(5)-C structure, rather than the usual C-x(6)-C-x(6)-C structure [10]. These new type of sequences were originally named “A2” because these sequences clustered with neither *AgRP* nor *ASIP* in the phylogeny. In 2006, another group (Kurokawa *et al*. [11]) reported for the first time the presence of four distinct Agouti genes in *T. rubripes* (torafugu) and these were termed *ASIP1*, *AgRP1* and *ASIP2*, *AgRP2*. Kurokawa reported differential expression of *AgRP2* in dorsal and ventral skin, indicating a role in pigmentation. Phylogenetic analysis suggested that the *ASIP2* and *AgRP2* or “A2” sequences clustered with *AgRP1*, hence indicating a higher similarity between the A2 (*ASIP2* and *AgRP2*) sequences with *AgRP* than *ASIP*. This paper also presented synteny evidence suggesting a relation between *AgRP1* and *AgRP2*. But unfortunately this synteny was based on only few genes that were placed on scaffolds that have all subsequently been changed [12]. The authors presented a theory that *AgRP2* came from AgRP1, and that the ASIP2 gene may have derived from the *ASIP1* gene. Based on this theory they introduced the present nomenclature of *AgRP2* and *ASIP2* for the “A2” genes and this nomenclature has been commonly used since.

Interestingly, the “A2” genes have a functional role in white background adaptation in zebrafish, mediated by *AgRP2* through direct optical sensing in the pineal gland [13]. The *AgRP2* peptide causes an increase in MCH peptides, and triggers a change in pigmentation by inducing pigment aggregation and most probably melanophore apoptosis [13]. It is shown that *AgRP2* acts on the MC1 receptor, thus making it in function *ASIP*-like. Evolutionary studies showed the presence of an *ASIP* like gene in a cartilaginous fish, *C. milii* (elephant shark), representing the most ancient version of an Agouti-like gene [14]. However, no Agouti-like sequences were found in the cephalochordate *B. floridae* (lancelet) or in lamprey, despite the fact that a functional MC receptor system exists in the sea lamprey [15].

Two concurrent letters to the editor appeared on the evolution of the Agouti-like genes in 2011 [12], [16]. Braasch and Postlethwait proposed that AgRP2 is an “ohnolog gone missing in tetrapods”, and that the A2 genes should be re-named *ASIP2a* (*ASIP2*) and *ASIP2b* (*AgRP2*). The authors postulated that the original Agouti gene underwent R1 (round one) of WGD (whole genome duplication), forming the proto-*AgRP* and *ASIP* genes. These proto-genes, in turn, underwent R2, forming two copies of each. The authors put forward an evolutionary model, where proto-ASIP, which was formed from proto-Agouti in R1, then duplicated again in R2, forming two lineages. One of these copies (proto-A2) duplicated in teleost-specifc genome duplication, giving rise to AgRP2 and ASIP2 (or ASIP2a and ASIP2b). Underpinning this argument, in addition to an phylogenetic tree, was use of a tool known as “synteny DB dotplots” [17], which can be used to visually inspect one-dimensional tracks showing the amount of synteny between a region of interest in one organism, and all chromosomes of another organism. Initially, the authors used this method to make the observation that *AgRP* in human has synteny similarity to *AgRP1* in zebrafish, while they observed that *AgRP2* did not share syntenies with *AgRP* in human. Braasch *et al*. then proceeded to look at data from *O. latipes* (medaka), and discovered a region in the human genome (Hsa 8 (60–100 Mb) that they found to contain three of Kurokawa’s original marker genes (*SNX16*, *WWP1*, and *RIPK2*). The authors assumed that they had found an ancestral “*A2*” area in human, lacking the actual *A2* genes, but preserving synteny with not only one, but both *A2* areas in fish. Then, using this alleged *A2* area, they proceeded to a comparison in human, noting a slightly higher degree of similarity between the *ASIP* synteny area in human and the Hsa 8 region, than between the *ASIP* synteny area in human and the *AgRP* synteny area in human.

We were allowed to present a short comment to these hypotheses in the same issue [12]. We showed that the choice of root in a maximum likelihood tree of the same set of Agouti-like sequences determines the positioning of the *A2* subtree in relation to the *A1* clusters within this dataset. We showed that if the phylogenetic tree was rooted on the elephant shark *ASIP* sequence, the oldest full-length sequence available, the *A2* sequences clustered with *AgRP*, not *ASIP*. This was originally shown by a low bootstrap value suggesting that the current sequences available were not sufficient to determine if the *A2* sequences were more similar to the tetrapod *AgRP* or *ASIP* sequences, which was one of the fundaments in Braasch and Postlethwait’s hypotheses. The common structural feature C-x(6)-C-x(5)-C of the teleost *A2* sequences and the phylogeny would however clearly suggest their common origin, in contrast to what was originally suggested by the Kurokawa nomenclature [11].

The functional importance of the *AgRP*, *ASIP* and the *A2* peptides, as well as the controversy about the evolutionary history of these sequences warrants further analysis. Here we present new Agouti sequences, and phylogenetic and structure modeling which are useful arguments for and against alternative evolutionary schemes. We also look further into the methods of determining synteny and implement a new method, the sinusoidal Hough transform [18], a pattern recognition technique previously used in microarray analysis (e.g. [19]) and other areas of image analysis in biology and medicine, as an interesting tool to detect linear synteny between two organisms.

We find fairly good agreement between the phylogeny, motifs and structural properties which supports the evolutionary events we suggest here. We do however not find specific synteny evidence that the *AgRP*, *ASIP* and *A2* genes could represent specific branches in a 2R duplication scheme. It is well known that many, if not most large chromosomal regions in teleosts, have synteny with one or many regions in the human genome. The fact that a teleost region, where one of the *A2* genes resides in, has synteny with humans does not validate a 2R duplication scheme. Moreover, such duplication scheme would require multiple losses of genes. There are also several other regions that are in synteny to this particular teleost region as well as for the teleost region where the other *A2* genes are placed. The presence of *ASIP2* in lobe-finned fish, as well as the absence of *AgRP2* in or near linear synteny blocks in gnathostome ancestor element regions 10, 3b, 7b, and 7c, suggests that the duplication of the synteny block containing the teleost *A2* genes may not have occurred in the 3R.

## Results

### 1. Database Annotation of A1 and A2 Sequences

We followed §10, §12, §16 of INSDC TPA policy, basing our A2 entries on pre-existing Agouti-like sequences entries by the same submission group, which include: (NP_001026628.1), (NP_001129.1), (CAH60801.1), (CAH60802.1), and (CAH60803.1). Details are given in Table S3.

### 2. Experimental Determination of European sea bass (*D. labrax*) *AgRP1*, *AgRP2*, *ASIP1*; Turbot (*S. maximus*) *ASIP1*; Solea (*S. senegalesis*) *ASIP1*

Reverse transcription-polymerase chain reaction (RT-PCR) using degenerate primers designed by alignments of available fish *ASIP1* or *AgRP1* sequences produced a partial cDNA fragments for sole and turbot *ASIP1* as well as sea bass *AgRP1*. The putative translations exhibited high identity with the C-terminal cysteine domain of the published *ASIP1*/*AgRP* sequences. To obtain the sequence of the complete peptide precursor RACE-PCR was performed in the 3′ and 5′ directions with specific primers. 3′ RACE generated unique bands for all three species and provided information about the coding region of the exon 4 and the 3′ untranslated region. 5′ RACE experiments also generated unique and provided information about the first exons as well as the 5′ untranslated region. The sea bass *ASIP1* and *AgRP2* sequence was obtained by blasting Genebank and Aquagenomics database, respectively with seabass *AgRP1* sequence. Subsequently, both sequences were cloned by RT-PCR and sequenced to corroborate data obtained *in silico*.

The peptide precursors have the same organization as other species. The poly-cysteine domain contains 10 cysteine residues with identical spatial pattern to that of Agouti-like proteins. Similar to mammalian *ASIP* molecules fish *ASIP1* sequences do not exhibit a short amino acid extension following the tenth cysteine residue as *sbAgRP1* and *sbAgRP2* do. All four peptides, fish ASIP1 and sea bass *AGRP1*, exhibits the cysteine knot structure *A1* i.e. C-x(6)-C-x(6)-CC whereas sea bass *AgRP2* shows the typical *A2*-like structure i.e C-x(6)-C-x(5)-CC.

### 3. Use of HMM to Search for Agouti-like Sequences

**Figure 1:**
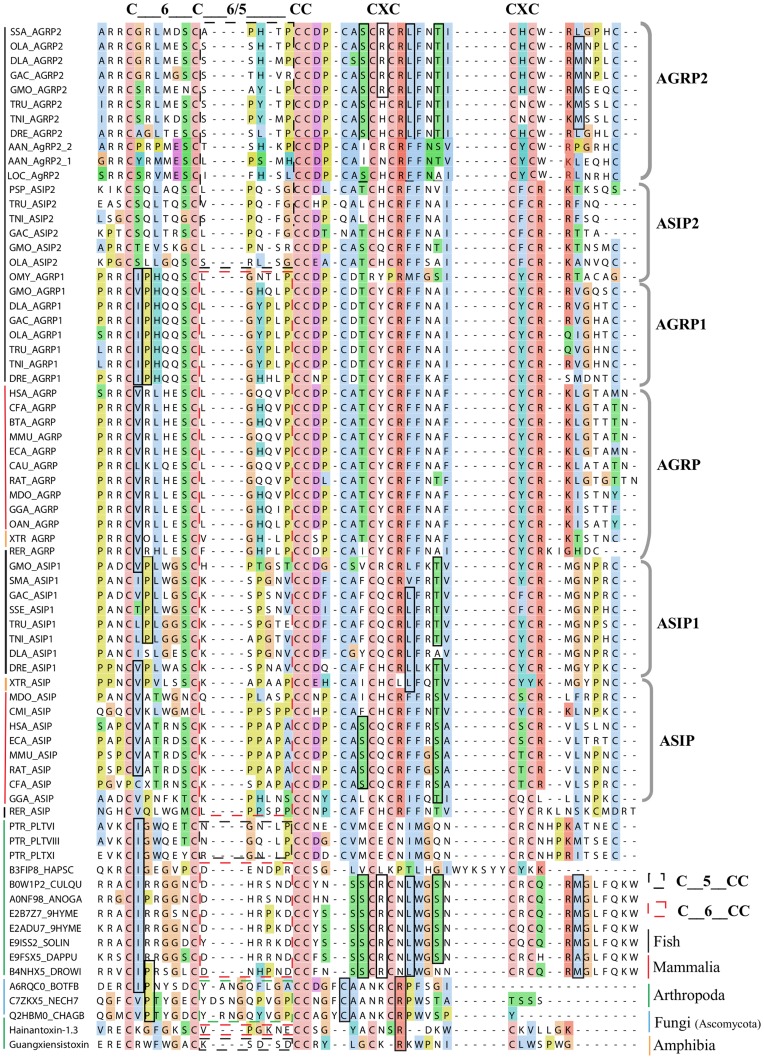
Multiple sequence alignment showing shared and group-specific motifs between the Agouti-like sequences. We include sequences that are previously published or already known for *AgRP* and *ASIP*-like clusters. The alignment includes the newly identified Agouti-like sequences in arthropods and in fungi. The important C-x(6)-C and the C-x(5,6)-C motif regions are shown above the alignment. The sequences that have the C-x(6)-C and C-x(5)-C motifs are distinguished with red and black dotted rectangular boxes, respectively. The residues that are conserved between the novel sequences and the different groups are shown with black rectangular boxes. The sequences that belong to different lineages are indicated with the colored line segments at the left of the alignment.

We searched for *AgRP* and *ASIP*-like sequences against the UniProt database restricted to a sequence length that range < = 150 residues. A search for homologues using the separate HMM profiles against our dataset (1,240,895 sequences; length < = 150 residues) significantly identified eight novel homologues from the phylum arthropods and three from the phylum ascomycota in the fungi kingdom. Multiple sequence alignment of the final dataset demonstrated that the novel sequences in the arthropods have the characteristic C-x(6)-C-C motif which are present in the *AgRP*, *AgRP1* and *ASIP*, *ASIP1* sequences. Furthermore, three sequences from fungi have longer C-x(8,9)-C-C motif instead of C-x(5,6)-C-C motif. Moreover, these three sequences have the C-[VI]-P motif and the C-A motif that are conserved in most of the *AgRP* and *ASIP*-like sequences. The conserved motifs between the novel sequences and the *AgRP* and *ASIP*-like sequences are shown in Figure 1.

### 4. PHI-BLAST Search of *A2*-like Sequences

In the PHI-BLAST 2.2.25+ search, the top hit for *AgRP2* is (XP_002937367.1) (*X. tropicalis AgRP*, which contains the motif C-x(6)-C-x(5)-C, despite being an *A1* sequence). The second best hit is a venom peptide from Mojave Desert spider (*P. tristis*), “Plt-VI” (AAC47205.1). The cysteine knot of Plt-VI (and also “Plt-VIII” or “Plt-XI”) is thus identical to *AgRP2* (C-x(6)-C-x(5)-C-C-x(2)-C-x(2)-C-x-C-x(6)-C-x-C-x(6,8)-C). Some spider toxin sequences are also similar (Table 1), in terms of cysteine knot structure, to Atlantic cod *ASIP2*. Spider toxin cysteine knots invariably start with C-x(6)-C. The next inter-cysteine segment varies in length from 5–7 amino acids (e.g., *C. guangxiensis* has 5, *O. hainana* has 6, and *A. orientalis* has 7). In the desert grass spider (*A. aperta*), this inter-cysteine segment is replaced by x(6)-C-x, giving a total length of 8, but that is an exception. Furthermore, all spiders have the C-C pair, followed by an inter-cysteine segment of length x(4,5). Only *P. tristis* has this segment punctuated by a single cysteine, making it much more *AgRP2*-like (but some *ASIP2* sequences actually lack this feature). The Eurasian yellow sac spider (*C. punctorium*), has 8 residues in this span, making it a highly exceptional structure. After this, only some spiders contain the paired C-x-C-x(n)-C-x-C feature, others only have C-x(n)-C, which is the case in the Chinese bird spiders, and also in tarantulas and in the King baboon spider. Finally, no spider, except *P. tristis*, contains the additional cysteine after the “paired” feature. The cysteine knot of torafugu *ASIP2*, C-x(6)-C-x(5)-C-C-x(5)-C-x-C-x(6)-C-x-C-x(7), is remarkable similar to a sequence from wolf spider (TXJ07_LYCSI), where the cysteine knot has the structure: C-x(6)-C-x(5)-C-C-x(4)-C-x-C-x(6)-C-x-C-x(14). The venom peptide Plt-VI displays many Agouti-like features: in terms of the length (130 amino acids), positioning in the sequence (at the end), and other sequence similarity with *AGRP1* (e.g. I-x(2)-Q in the first inter-cysteine segment, G-x(1,2)-L-P in the second segment, as well as one or two cysteines in the beginning of the sequence, before the actual inhibitor knot).

**Table 1:** Cysteine knots in spider toxins.

Species	Cysteine knot structure
Tarantula (*H. schmidti*)	C-x(6)-C-x(6)-C-C-x(4)-x(14)-C
Funnel web spider (*A. robustus*)	C-x(6)-C-x(5)-C-C-x(3)-C-x(13)-C
King baboon spider (*C. crawshayi*)	C-x(6)-C-x(5)-C-C-x(4)-C-x(6)-C
Chinese bird spider (*O. hainana*)^1)^	C-x(6)-C-x(6)-C-C-x(4)-C-x(6)-C
Bird spider (*C. guangxiensis*)^2)^	C-x(6)-C-x(5)-C-C-x(4)-C-x(6)-C
Funnel spider (*A. orientalis*)	C-x(6)-C-x(7)-C-C-x(4)-C-x-C-x(5)-C-x-C
Yellow sac spider (*C. punctorum*)	C-x(6)-C-x(6)-C-C-x(8)-C-x-C-x(8)-C-x-C
Wolf spider (*L. singoriensis*)	C-x(6)-C-x(5)-C-C-x(4)-C-x-C-x(6)-C-x-C
ASIP2 (teleost-typical)	C-x(6)-C-x(5)-C-C-x(5)-C-x-C-x(6)-C-x-C
Desert grass spider (*A. aperta*)	C-x(6)-C-x(6)-C-x-C-C-x(5)-C-x-C-x(10)-C-x-C
Mojave Desert spider (*P. tristis*)	C-x(6)-C-x(5)-C-C-x(2)-C-x(2)-C-x-C-x(6)-C-x-C-x(8)-C
AgRP2 (teleost-typical)	C-x(6)-C-x(5)-C-C-x(2)-C-x(2)-C-x-C-x(6)-C-x-C-x(6)-C

1) The species that contains Hainantoxin-1.3.

2) The species that contains Guangxiensistoxin.

### 5. Bayesian Phylogenetic Analysis of A1, A2, and Agouti-like Sequences

**Figure 2:**
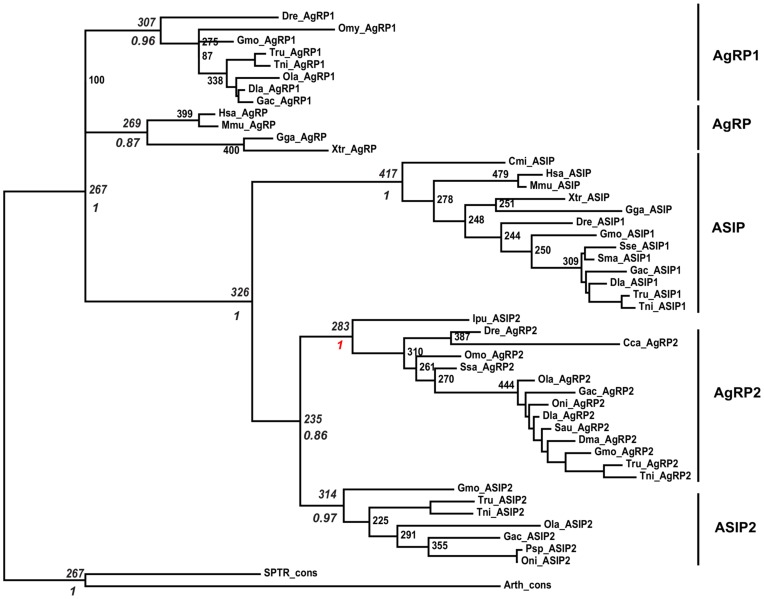
Phylogenetic analysis of Agouti-like sequences. The unrooted maximum likelihood phylogenetic tree of Agouti-like sequences. Robustness of the nodes is tested with the posterior probabilities based on MCMC analysis as implemented in the MrBayes program. Both bootstrap (out of 500 replicates) and the posterior probability support were given in italics for the significant nodes. The posterior probability (1) of the A2 node is highlighted in red color. The tree is rooted on the node that clustered the consensus sequences, which serves as out-group.The tree shows that *A2* is likely to have branched from *ASIP1*.

The phylogenetic relationship of the Agouti-like sequences was investigated using the Bayesian approach as implemented in MrBayes 3.1.2. The topology supported by the Bayesian approach was also verified using the Maximum Likelihood approach as implemented in PhyML 3.0. We constructed several preliminary trees to test the robustness of the diversification of the Agouti-like sequences particularly when the tree is rooted. In order to check the most stable topology supported by the root, we made three separate consensus sequences using HMMEMIT (see Methods), one with the sequences identified in spider (SPTR_cons), second with sequences identified in arthropods, excluding the spider sequences (Arth_cons) and third as combined together (Arth1_cons). Except for the tree rooted on Arth1_cons, all the trees clustered *AgRP*/*AgRP1* together basal to the root and clustering *AgRP2 ASIP2* and *ASIP* together (100%). Considering the most stable topology supported by preliminary trees, the tree was finally rooted on consensus sequences SPTR_cons and Arth_cons (see Figure 2) that clustered *ASIP*, *AgRP2* and *ASIP2* together (100%) and separating *AGRP* and *AgRP1* clusters basal to the root.

### 6. Structure Modeling of *A2* Sequences

**Figure 3:**
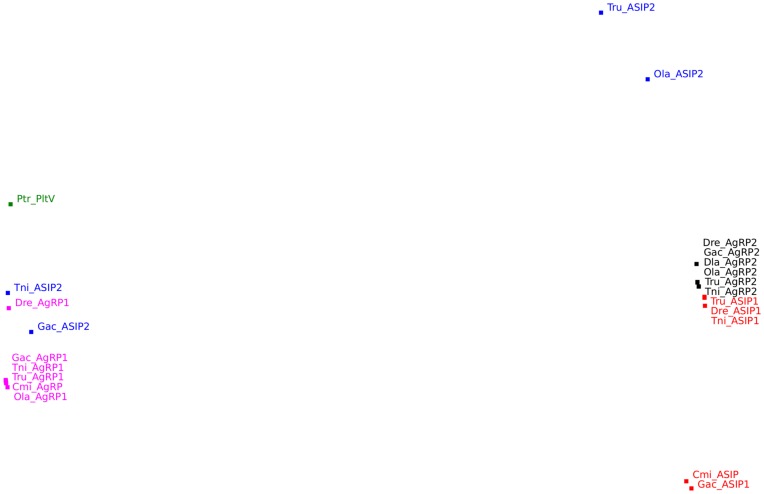
Two-dimensional representation of the similarity relationships between the structure models of A1 and A2 inhibitor cysteine knots, as obtained by non-metric multidimensional scaling. The MDS fit measures (s-stress  = 0.14, RSQ = 0.95) indicate that the inter-model distances in this configuration reflect well the original inter-model dissimilarity values. The figure is generated by a Perl script that outputs support vector graphics. The figure shows that AgRP2 is more ASIP-like, and ASIP2 more AgRP-like, The shift of the A2 points towards ASIP agrees with the Mr. Bayes phylogram. Ptr Plt-VI is more AgRP-like.

Non-metric multidimensional scaling was used (see Materials and Methods) to construct a two-dimensional representation of the similarity data, in which the data points are positioned so that the distances between them reflect as much as possible the original dissimilarity values (Figure 3). The resulting configuration shows a clear bipartitioning of the *AgRP* or *ASIP* structures. Notably, the Mojave Desert spider venom peptide Plt-VI sequence falls within the range of the other Agouti-like sequences.

In human, both *AgRP* and *ASIP* have 10 cysteines that hold the knot together. Starting from the N-terminal end, the sequence passes the first cysteine, which holds together the first loop, which is shortened by one residue in *AgRP2*. The first loop has two disulphide connectors with the active site loop, which contains the R-F-F motif. The arginine (R) residue is large and basic, giving a rotamer with large conformational potential. In *AgRP*, the active site loop contains two small anti-parallel beta sheets, and an internal disulphide connector between the beta sheets. This disulphide bond is missing in many venom peptides. The R-F-F motif is placed on the N-terminal side of the active site loop, directly after the first beta sheet in the active site loop. The disulphide connectors between the first loop and the active site loop cross over each other, i.e. if the structure is viewed from top/down, looking towards the two loops from top, the disulfide bonds holding the two loops together from a cross. After leaving the active site loop, there is a final cysteine holding the C-terminal chain together with the peptide sequence that connects the two loops.

The *AgRP* structure is similar to many known structures, including a triple beta sheet containing gene product (1×I7) from polydnavirus, a virus which is transmitted during oviposition of parasitic wasps. Other similar known structures include: sea anemone toxin (1ANS), *A. aperta* calcium channel blocker (1AGG), *A. dohrni* assassin bug saliva calcium channel blocker (1LMR), plant sweet taste perception blocker (1C4E), central Asian spider P2×3 pain receptor blocker (2KGU), and palutoxin (a sodium channel blocker). The greatest difference between *AgRP* and these toxins to which it shows high similarity, is the absence of the disulphide bond connecting the beta sheets, as well as the absence of the disulphide bond holding the C-terminal chain more closely to the knot structure. Plt-VI, despite being a spider venom peptide, has 10 cysteines, including the disulphide connector between the beta sheets, and the disulphide connector holding the C-terminal chain close to the knot.

Because *AgRP2* and *ASIP2* have a shortening of the first loop by one residue (C-x(6)-C-x(5)-C, instead of C-x(6)-C-x(6)-C), we wanted to know if this would affect the positioning of the beta sheets or the active site. We considered the possibility that the shorter first loop in *AgRP2* could result in a re-positioning of the active site or the beta sheets. Because the C-x(6)-C-x(6)-C structure is one residue longer, we postulated that the peptide sequence might buckle out more than the C-x(6)-C-x(5)-C variant. In the structure model of Plt-VI, we noted a shortening of the beta sheets in the active site loop, possible a result from strain in the loop pulling the sheets apart. On the other hand, in *ASIP2*, we noted the possibility of a third beta sheet in the affected first loop, showing hydrogen bonding potential between the beta sheets in the active site loop and the first loop.

### 7. Use of a Sinusoidal Hough Transform to Search for Linear Synteny between Human Chromosome 8, Region 60–100 Mb, and Medaka Chromosomes 17 or 20

Medaka chromosomes (17 and 20) contain linear synteny (i.e. a continuous block of orthologues in 2-dimensional scatter plots of gene coordinates) with human chromosome 8, region 60–100 Mb. Compared with medaka chromosome 17, medaka chromosome 20 contains almost twice (44 compared with 25) as many orthologues with the human chromosomal region, and the proportion of these lying in the largest linear synteny block has increased from 44% (11/25 on Ola 17) to 64% (28/44 on Ola 20) (see Table 2). The angle stated for (θ) is the highest (or furthest away from the horizontal plane) of the range of angle bins that reach the stated level of (“S”). In this table (Table 2), a filter is used to divide any clusters that contain a gap larger than 5,000,000 basepairs. The remaining 22 medaka chromosomes that are not listed contain fewer than two orthologues with the area of interest in the human genome, and are hence not listed. The interpretation of this result is that the synteny relationship between the recently proposed, ancestral A2 area in the human genome (Hsa 8, 60–100 Mb) and medaka chromosomes 17 (containing *AgRP2*) and 20 (containing *ASIP2*), differs both in the amount of orthologues, and in the amount of orthologues placed in the largest linear synteny block. A two-dimensional plot of gene start coordinates of orthologue pairs between the area on Hsa 8 (60–100 Mb) and Ola 17 and Ola 20 illustrate the difference: while Ola 17 only contains some small (containing 11 genes, or less) islands of synteny, the Ola 20 plot (data not shown) contains a large conserved cluster of 28 genes.

**Table 2:** Hough transform comparison of synteny between Hsa 8, 60–100 Mb, and Ola 17 and 20.

Chromosome	Orthologues	θ	ρ	Biggest linear synteny block (“S”)
11	19	45.50	80.55	3
16	24	55.50	83.15	8
17	25	37.50	46.85	11
20	44	13.50	15.45	28

### 8. Evaluation of the Background Frequency of Randomly Placed 40 Mb-sized Windows from the Human Genome that Contain More Synteny with Medaka Chromosome 17 or 20, than Human Chromosome 8, Region 60–100 Mb

**Figure 4:**
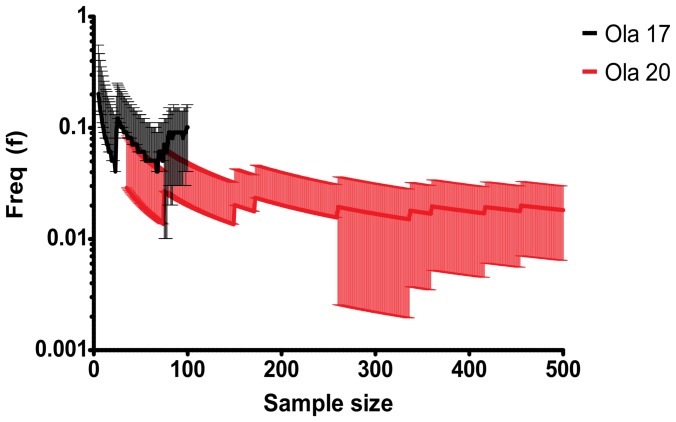
Visual representation of the sampling process of the human genome. Visual representation of the sampling process of the human genome, where the x-axis represents the current sample size and the y-axis the frequency of windows that are characterized as “positive” hits against either Ola 17 or Ola 20. For each placed 40 Mb-sized window, we characterize the window as being a positive hit to either medaka chromosome 17 or 20, if it contains more orthologues (more synteny) with Ola 17 or Ola 20, and human chromosome 8, region 60–100 Mb. The error bars represent a 95% confidence interval that depends on the sample size; if the lower limit of a 95% confidence interval is negative, it is shaded on the mean. The sampling process was terminated at N = 100 and N = 500, for medaka chromosome 17 and 20, respectively. The mean frequencies and 95% confidence intervals are, at the termination points: f = 0.1000 for medaka chromosome 17 (95% CI: 0.0412−0.1588); f = 0.0180 for medaka chromosome 20 (95% CI: 0.0063−0.0297). We use a log scale for ease of reading.

To obtain a statistical assessment of the proposed [16] ancestral *A2* area in the human genome, we used a sliding window method, where a 40 Mb-sized window was placed repeatedly at random locations in the human genome, however not allowing any overlap with the area Hsa 8, 60–100 Mb. A window was characterized as a “positive” hit, if it was found to contain at least as many orthologues in synteny (meaning orthologues placed in the same regions, but not necessarily clustered in a 2-dimensional scatter plot of gene start coordinates), for Ola 17 or Ola 20, respectively. By repeated sampling, we could calculate a 95% confidence interval of the frequency of obtaining a positive hit for either Ola 17 or Ola 20, which depends on the sample size. The sampling was carried out until a confidence interval had stabilized (see Figure 4). The termination points for Ola 17 and Ola 20 was N = 100 and N = 500, respectively. The confidence intervals of the frequency of “positive” hits for Ola 17 and Ola 20 was the following: f = 0.1000 for medaka chromosome 17 (95% CI: 0.041−0.16); f = 0.0180 for medaka chromosome 20 (95% CI: 0.0063−0.030). The final proportions can be recalculated as exact binomial confidence intervals using R 2.13.2 1-sample proportions test with continuity correction: f = 0.1000 for medaka chromosome 17 (exact binomial 95% CI: 0.052−0.18); f = 0.0180 for medaka chromosome 20 (exact binomial 95% CI: 0.0088−0.035). This means that the probability for a randomly placed window in the human genome to display an as large amount of synteny as in the comparisons between Hsa 8 (60–100 Mb and Ola 17 or Ola 20, is about 10% for Ola 17, but less than 2% for Ola 20. This highlights a statistical difference between these medaka chromosomes in their similarity with the proposed ancestral area in the human genome.

### 9. A Control Experiment to Test Degree of Clustering on Medaka Chromosomes 17 and 20, of Orthologues Located in the Region Hsa 8, 60–100 Mb

To investigate whether it would be possible to use “synteny DB dotplots” to test suspected findings, such as the proposed ancestral area [16], we devised a control experiment. The purpose of the control experiment was to determine if the alleged ancestral area indeed represented a clustering of genomic coordinates in both query and target organisms. Thus, we attempted to reverse the experiment shown in panel “C” in Braasch *et al*., by using the *O. latipes* chromosomes as query, and Hsa 8 as target.

The dotplots of medaka 17 and 20, this time used as query chromosomes against full-length Hsa 8, show a striking difference in gene density, where the linear synteny area on medaka 20 now manifests as a clearly visible cluster (located at 14–15.5 Mb in Ola 20). Medaka chromosome 17, on the other hand, shows no comparable high density area. Because no particular region can be specified for chromosomes displayed on the y-axis in synteny database dotplots, many of the points will represent genes not located in the relevant region on Hsa 8. Furthermore, the visualization method has no filter to identify linear synteny, or the largest linear synteny block (“S”), as opposed to closely spaced orthologs.

### 10. Synteny Dotplot Results Indicate that Multiple Regions in the Human Genome are Syntenic with the AgRP2 and ASIP2 Regions in Teleosts

**Figure 5:**
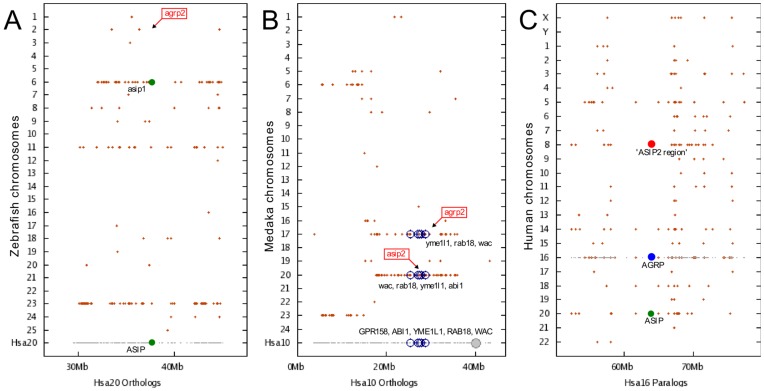
Conserved synteny dot plots derived from the Synteny Database [**17**]. (A–C) Conserved synteny dot plots derived from the Synteny Database [17]. (A) The zebrafish *AgRP2* region on Dre 2 (red box) shares conserved syntenies with neither the zebrafish *ASIP1* region (Dre 6) nor with the human *ASIP* region (Hsa 20). (B) The *AgRP2* and *ASIP2* regions in medaka and other teleosts share conserved synteny with each other and with a region on human Hsa10, including several *AgRP2*- and *ASIP2*-neighboring genes. (C) Analysis of the human genome shows that the *AgRP* region on Hsa16 shows more paralogous connections to the inferred *ASIP2* region on Hsa 8 than to the ASIP region on Hsa 20 (183 vs. 82 genes, respectively, not restricting Y-axis chromosomal regions).

Our experimentation confirms the previous result that the teleost *AgRP2* chromosomal region shares syntenies neither with the teleost *AgRP1* region nor with the tetrapod *AgRP* region [16]. Furthermore, our investigation of this gene family using synteny data clearly indicates that the teleost *AgRP2* chromosomal neither shares syntenies with the teleost *ASIP1* region nor with the tetrapod *ASIP* region (Figure 5, Panel A). Therefore, the teleost *AgRP2* chromosomal does not share any syntenies with the *AgRP* or *ASIP* regions in teleosts or tetrapods.

We can also confirm the previous result [16] that teleost *AgRP2* and *ASIP2* regions show conserved synteny to a region on human chromosome 8. However, our experimentation shows that this is not the only ancestral region of interest in the human genome. For example, we have identified an area on human chromosome 10 (Hsa 10) (Figure 5, Panel B). The Hsa 10 area (3–43 Mb) shown contains 48 orthologues in synteny with Ola 20 (*ASIP2*) and 26 orthologues in synteny with Ola 17 (*AgRP2*), the highest recorded number of orthologues in synteny with Ola 20 in the human genome. This is comparable to the corresponding result for the Hsa 8 area (44 and 25 orthologues, respectively) [16]. A set of markers from Hsa 10 are shown (Figure 5, panel B): *GPR158*, *ABI1* (Ola 20), *YME1L1* (Ola 17/20), *RAB18* (Ola 17/20), *WAC* (Ola 17/20). *RAB18* is located 2.1 Mb from *ASIP2*. There are several areas in the human genome that exceeds the Hsa 8 (60–100 Mb) area for amount of synteny with Ola 17, such as Hsa 19 (1–41 Mb), containing 58 orthologues with Ola 17. Analyzing the Hsa 10 region with the Hough transform shows that this area contains 8 and 14 orthologues (with Ola 17 and Ola 20, respectively) in linear synteny blocks, a result that can be compared with 11 and 28 orthologues, for the Hsa 8 area. This difference indicates that the Hsa 8 area is highly syntenic with Ola 20. In fact, only one other area in the human genome, at chromosome 3 (110 Mb–150 Mb) is detected as containing more (30 orthologues in linear synteny block) with Ola 20. Other areas containing > = 11 orthologues in linear synteny with Ola 17 are found using the Hough transform at approximately 10% of randomly placed genomic windows in the human genome.

It is true that the previously identified regions [16] in the human genome – Hsa 16 (*AgRP* region), Hsa 8, and Hsa 20 (*ASIP* region) – most likely derived from a single *ASIP*/*AgRP* region on the ancestral vertebrate proto-chromosome B. Furthermore, Hsa 10 (3–43 Mb) contains 48 and 26 orthologues in synteny with Ola 20 and Ola 17, respectively. Hsa 19 (1–41 Mb) contains 58 orthologues with Ola 17. Using the Hough transform, we discovered an area on Hsa 3 (110–150 Mb) which contains more than 30 orthologues in linear synteny with Ola 20. Thus, there are at least six regions in the human genome that indicate strong syntenies to the regions containing *AgRP2* and *ASIP2*. The *AgRP* region in the human genome contains more (183 vs. 82 genes) syntenies with the chromosome (Hsa 8) containing the inferred *ASIP2* region than the chromosome containing the *ASIP* region in human (Figure 5, Panel C).

Considering the reconstruction data of the early vertebrate and gnathostome karyotypes [20], it is clear that medaka chromosome 17 contains the same gnathostome ancestor chromosome elements as medaka 20∶10, 3B, 7B, 7C. In addition, medaka chromosome 17 contains the following gnathostome ancestor chromosome elements: 1a, 1c, 19a, and 19c. The area on Hsa 10 where we found synteny with both the *AgRP2* and *ASIP2*, contains the gnathostome ancestor chromosome 10 elements, and the area on Hsa 19 that we found to have synteny with Ola 17 contains 19a, 19b, and 19c. The region on medaka chromosome 3, containing *AgRP1*, derives from a completely different region, the gnathostome ancestor chromosome element 15. Among the previously identified putative synteny regions and the ones that we have identified here (see above) that contain Agouti-like areas in the human genome (ie Hsa 3, Hsa 8, Hsa 10, Hsa 16, Hsa 19, Hsa 20), it is only Hsa 8 and Hsa 16 that are convincingly derived from ancestral vertebrate proto-chromosome B which is found in the amniote and osteichthyan ancestor. The other areas are more likely related to J (proposed Hsa 3 area), E and D (Hsa 10 area), and A (Hsa 19 area). The regions containing the A2 genes in medaka are not only related to proto-chromosome B, but also to A, E, and F. In Figure S2, it can be seen that of the 80 same-name orthologues that exist between Ola 17 and Ola 20, there are 3–4 linear synteny blocks in the region of gnathostome ancestor elements 10, 3b, 7b and 7c. However, our genes of interest, *AgRP2* and *ASIP2*, are not located in or near any such blocks.

Our efforts to trace A1 and A2 markers in teleosts and sea lamprey can be found in the online material (Tables S1 and S2).

### 11. Search for A2-like Sequences in Little Skate, Spotted Gar, and European Eel

In little skate, using build 2, we found one target sequence on contig LSb2-ctg674736 (1474 - 1331). However, using build 1, we found an additional target sequence: LER_WGS_1_CONTIG_1088548. Both of the sequences have the C-x(6)-C-x(6)-C form, and the R-F-F form of the functional motif. No A2-type sequences are found in this organism.

We were able to locate the full-length ASIP, on the following contigs: 1656154/AESE011535652 (start of the sequence), 1715056/AESE011594554 (middle exon), and 1088548/AESE011079059 (the cysteine knot).

In spotted gar, we found one A2-like sequence on AHAT01017486.1, and we TPA annotated this finding as: BR000972. The contig contains ATP6V0D2, an AgRP2 marker in teleosts. The sequence has the R-F-F form of the functional motif, and the the C-x(6)-C-x(5)-C form of the cysteine knot. The sequence contains the middlemost and last cysteines (AgRP2 feature). Spotted gar also contains the normal AgRP and ASIP.

In European eel, we found four scaffolds that contain Agouti-like genes: scaffold9054, scaffold1167, scaffold3173, scaffold1776. Two of these (on scaffolds 9054 and 1167) sequences have the C-x(6)-C-x(5)-C form of the cysteine knot, and both contain the the R-F-F form of the functional motif. For the 9054 scaffold, we were able to use GenScan to find a 3 exon full-length sequence. One of the A2 sequences in eel apparenly lacks the last cysteine.

## Discussion

The lack of sequences has hampered studies of the evolution of the Agouti-like peptides. We therefore expanded the sequence pool of vertebrate Agouti-like sequences, which in turn enabled us to develop a sensitive, profile-hidden Markov model for long-range searches. Using these models and PHI-BLAST searches, we surprisingly found eight novel homologues from the phylum arthropods and three from the phylum ascomycota in the fungi kingdom that have similarities with Agouti-like peptides. Importantly, the sequences in arthropods have the characteristic C-x(6)-C-C motif which are present in the Agouti-like sequences. The three sequences from fungi have however the longer C-x(8,9)-C-C motif instead of the C-x(5,6)-C-C motif but these three sequences have also the C-[VI]-P motif and the C-A motif that are conserved in most of the AgRP and ASIP-like sequences. These sequences do not only share these characteristic motifs (see Figure 1) but they also have the approximate sequence length (about 130 amino acids), and positioning of the motif sequence, i.e. in the end, that matches the vertebrate Agouti-like sequences. Moreover, we found that two of the sequences, one from the African malaria mosquito (*A. gambiae*, A0NF98) and another one from the Southern house mosquito (*C. quinquefasciatus*, B0W1P) share exon-intron structures that are similar to the other Agouti-like peptides (online appendix, Figure S1), providing further support for the common origin of these sequences. The sequence that is the most similar to any vertebrate Agouti-like sequence is a venom peptide from Mojave Desert spider (*P. tristis*) that contains a cysteine knot identical to *AgRP2*. It is unlikely that these Agouti-like sequences in arthropods or in fungi are functioning through MC receptors as the most ancient evidence of the MC receptors is found in sea lamprey [15]. Moreover, inhibitor cystein knot (ICK) structures in spider venom peptides are thought to give the proteins stability and protection against proteases, and in spiders, ICK proteins are known to block ion channels, not GPCRs [21].

We made a large effort to annotate a number of vertebrate Agouti-like sequences. We have added more than twice as many new A2 sequences from different sources such as from mining of new genomes (Atlantic cod *AgRP2* and *ASIP2*, and tilapia *AgRP2* and *ASIP2*), experimental sequencing (European sea bass *AgRP2*), and by using ESTs imported from Kurokawa *et al*. [11], [22] (African cichlid *ASIP2*, trout *AgRP2* and salmon *AgRP2*). The new sequences give us a more complete view of which sequence motifs, and which exon-intron structures, are typical of *A2* sequences. The larger *A2* sequence pool has allowed us to identify a new *A2* motif, present in the second coding exon of all known *A2* sequences, “L-F-A-R” (identified using Multiple ‘Em’ for Motif Elicitation). Furthermore, in the *A2* cysteine knot (which starts with C-x(6)-C-x(5)-C, not C-x(6)-C-x(6)-C), we show that the R-L-F motif is indicative of the sequence being *AgRP2*, and R-F-F of *ASIP2*. Otherwise the R-F-F is normally indicative of *AgRP1* (and R-L-F of *ASIP1*) in teleosts, in contrast to the current names *AgRP2* and *ASIP2*, but the change from R-F-F to R-L-F can be accomplished by a single nucleotide change.

Then we performed phylogenetic analysis and 3D structural modeling of these sequences. The arthropod and fungi sequences do not show a phylogenetic relationship to any of the specific sub-branches of the Agouti-like sequences (i.e. *AgRP*, *ASIP* or *A2*) but group in a special branch outside of the vertebrate tree (data not shown). However, the non-vertebrate sequences provide a very good root for the vertebrate tree, in line with the “ancestral” character of the sequences. The phylogenetic analysis shows that the *AgRP* sequences cluster basally in the tree, suggesting that these sequences split from a cluster containing both the *ASIP* and the *A2* sequences. Later the *ASIP* and *A2* split, and then the *A2* split into the *AgRP2* and *ASIP2*. This is in good agreement with the phylogeny presented previously by Braasch *et al*. [16], Kurokawa [11] and us [12]. The suggestion that *AgRP* is the most ancient of these branches and that *ASIP* is more closely related to *A2* is also supported by the intron structure of *AgRP*, which is much more compact than the one of *A2* or *ASIP*. It seems without a doubt that the *AgRP2* and *ASIP2* peptides have a common origin. This conclusion is also supported by our structural modeling. Protein structure prediction is generally not considered an alternative to resolving phylogenetic problems [23]. In this case, however, because the cysteine knot structure is highly conserved and structurally constrained by the disulfide bonds, the influence the interspersed residues can be modeled with a higher accuracy than many other structures. By limiting the modeling exercise to the cysteine knot region only, we obtained a set of theoretical structure models that could be compared by structure superposition, and root-mean square deviation (RMSD) comparison. The resulting set of pairwise RMSD distances could be analyzed using multidimensional scaling in the statistics package SPSS 17.0, obtaining a clustering where the RSQ (>0,87) and S-stress (<0,18) indicators showed good clustering. The multidimensional scaling showed that *AgRP2* and *ASIP2* are fairly similar, while the *AgRP* and *ASIP* clusters are most dissimilar. Interestingly the Agouti-like sequence from the Mojave Desert spider fell within the distances of the structures from the vertebrate Agouti-like peptides, providing further support to the conclusion that the arthropod sequences share a common origin with the vertebrate ones.

Synteny analysis of large chromosomal regions is difficult for many reasons: there is a lack of reliable tools that provide an objective measurement of synteny, certain synteny always occurs at random, most synteny regions contain genes that are not duplicated by block duplications events, and the objectivity of synteny of few genes among many can be questioned. At the same time, synteny is a unique way to establish how genes or chromosomal regions may have been copied through evolution. Here we used a new approach to look at the statistical evidence for synteny, a sinusoidal Hough transform pattern recognition technique that is able to detect co-linearities of points in two dimensions (see Materials and Methods). We compared the number of orthologues between all permutations of teleost chromosomes (from the species Dre, Gac, Ola, and Tni) containing Agouti genes (online appendix, Table S1), and noted a higher amount of synteny between *A2* containing chromosomes, contrasted to *A1* chromosome comparisons. Using a large sample of 40 Mb-sized human genomic windows, we found that there is a high difference in the probability of encountering a comparable amount of synteny between the medaka chromosomes 17 (*AgRP2*) or 20 (*ASIP2*) and the Hsa 8 (region 60–100 Mb), being 10% for the comparison with medaka chromosome 17 to only 2% for the comparison with medaka chromosome 20 (see Figure 4). These results challenge the conclusion of Braasch *et al*., because we find no evidence of a comparable, and significant amount of synteny to both the medaka chromosomes as suggested by Braasch *et al*. [12]. While our analysis confirms the synteny of Hsa 8 with teleost *AgRP2*, our experimentation shows that this is not the only ancestral region of interest in the human genome. For example, we have identified an area on human chromosome 10 (Hsa 10) [See Fig. 5, Panel B]. The Hsa 10 area (3–43 Mb) shown contains 48 orthologues in synteny with Ola 20 (*ASIP2*) and 26 orthologues in synteny with Ola 17 (*AgRP2*), which has the highest recorded number of orthologues in synteny with Ola 20 in the human genome. This is comparable to the corresponding result for the Hsa 8 area with 44 and 25 orthologues, respectively (Braasch *et al*.) [16]. The figure shows a set of markers that are present on Hsa 10, including: *GPR158*, *ABI1* (Ola 20), *YME1L1* (Ola 17/20), *RAB18* (Ola 17/20), *WAC* (Ola 17/20), where *RAB18* is 2.1 Mb from *ASIP2*. There are also several areas in the human genome that exceed the Hsa 8 (60–100 Mb) area for amount of synteny with Ola 17, such as Hsa 19 (1–41 Mb), containing 58 orthologues with Ola 17. Analyzing the Hsa 10 region with the Hough transform shows that this area contains 8 and 14 orthologues with Ola 17 and Ola 20, respectively, in linear synteny blocks, a result that can be compared with 11 and 28 orthologues, for the Hsa 8 area. Other areas containing > = 11 orthologues in linear synteny with Ola 17 are found using the Hough transform at approximately 10% of randomly placed genomic windows in the human genome. Moreover, using the Hough transform, we discovered an area on Hsa 3 (110–150 Mb) which contains more than 30 orthologues in linear synteny with Ola 20. Thus, there are at least six regions in the human genome that indicate some syntenies to the regions containing *AgRP2* and *ASIP2*. It is well established that medaka chromosomes 17 and 20 derive partly from gnathostome chromosomal elements 19c and 10 [20] that among other genetic elements, in turn may be part of a vertebrate proto-chromosome B. The origin of this region is much older than any trace of vertebrate Agouti-like peptides. However, we do not see any evidence that the *ASIP* and *AgRP* are found in any of the regions that are related to these regions in humans, which is generally considered to be the prerequisite for genes that have their origin in the tetrapliodizations events like the Hox genes [24], [25].

We find that while there is synteny imprint evidence for a common origin of A2 genes (for example, Ola 20 (*ASIP2*) shares >80 orthologues with Gac III (*AgRP2*)), much less points to a common origin of *AgRP1* and *AgRP2* (only 1–15 orthologues shared between relevant chromosome pairs). Recently, the sea lamprey genome was moved from “Pre ENSEMBL” (on September 16, 2011). The assembly into contigs of up to >1 Mb presents a new opportunity for us to trace the conservation of the synteny pattern prior to the teleosts. Interestingly, we found that in lamprey, there are 15 contigs that exclusively link *AgRP2* and *ASIP2* (see e.g. GL483536 or GL476773 in online appendix; Table S2), but only 7 contigs that exclusively link *AgRP1* and *AgRP2*. These results provide further support for a block duplication of the region containing *AgRP2* and *ASIP2*. Importantly, lamprey split from the lineage leading to the tetrapods before the 2R and surely before the 3R suggesting that the split of *AgRP1* and *AgRP2* did not happen through the two (or three) rounds of genome duplications.

Overall, our specific chromosomal region analysis in this study suggest that we are not able to find synteny imprints that would support that the A2 genes would have a specific synteny in the human genome, a key evidence for the hypothesis about the evolution of the Agouti genes presented by Braasch *et al*. This is in sharp contrast to many well-documented cases, which have gone from 1 to 2 to 4 to 8, minus some losses, including Hox [26], ion channels [27], opioid receptors [28], neuropeptide Y receptors [29], IGF-BP [30], and the endothelin system [31]. It is difficult to prove that the evolution could not have occurred according to the scheme that is presented by Braasch *et al*. However, we find this scheme very unlikely due to the following reasons: 1) The synteny analysis does not provide evidence that *ASIP*, *AgRP* and *A2* genes are three arms that resulted from a whole genome duplications (2R), 2) The scheme suggests that there are losses of four major branches, i.e. ohnologs gone missing from R2 duplication of ancestor to *AgRP*, OGM form tetrapod *A2* duplication as well as 3R genes for *ASIP1* and *AgRP1*, 3) The conserved synteny found in lamprey as well as the finding of *ASIP2* in lobe finned fish (coleacanth, Sarcopterygii), a linage that split from the lineage leading to mammals, after the split of teleosts, suggests that the A2 genes existed before 3R, 4) The absence of Agouti 2-like sequences in Chondrichthyes, such as the spiny dogfish does not support the conclusion that the 2R are important for the creation the Agouti peptide family.

In conclusion, here we provide a comprehensive analysis of the current set of Agouti-like sequences. There is considerable evidence that we have found Agouti-like sequences beyond the vertebrate kingdom. It is possible that these sequences originated through horizontal gene transfer, but this remains to be determined. We find that while it is theoretically possible that the evolutionary model proposed by Braasch *et al*. describes the order of events within the Agouti peptide evolution, the scenario is very improbable. We find that more likely, Agouti-like peptides, like most vertebrate gene families, were formed through classical subsequent gene duplications where the *AgRP* is likely to be the most ancestral, first splitting from a common ancestor to *ASIP* and *A2* and then later the *A2* split from *ASIP* followed by a split resulting in *ASIP2* and *AgRP2*. The finding of a single copy of AgRP2 in spotted gar and double copies of A2 in European eel appear consistent with a 3R origin, but the position of the AgRP2 and ASIP2 genes outside linear synteny blocks on their respective TSGD-duplicated chromosomes in Medaka could suggest a random copying event into the TSGD chromosomal context.

## Materials and Methods

### 1. Database Annotation of A1 and A2 Sequences

Please refer to the online appendix (Table S3), for details.

### 2. Experimental Determination of European Sea Bass (*D. Labrax*) *AgRP1*, *AgRP2*, *ASIP1*; Turbot (*S. Maximus*) *ASIP1*; Solea (*S. Senegalesis*) *ASIP1*

Turbot (HE598752) and sole (HE598753) *ASIP1* and sea bass *AgRP1* (HE660086) cDNAs were cloned by RT-PCR using degenerate primers followed by RACE-PCR. Sea bass *ASIP1* sequence (FM021895) covering the full coding sequences were obtained from NCBI blasting against expressed sequence tags (EST) database. Specific primers were then designed to amplify the sequence that was cloned into pGemT easy vector and sequenced. Sea bass *AgRP2* sequence (HE660087) was obtained from restricted access Aquagenomic databases (http://www.aquagenomics.es). As before specific primers were designed to clone and verify sea bass *AGRP2* sequence.

### 3. Use of HMM to Search for Agouti-like Sequences

We constructed separate HMMs (hidden Markov models) for *AgRP*, *AgRP1*, *AgRP2* and for *ASIP*, *ASIP1* and *ASIP2* clusters using the HMMER3 software. These separate HMM models were used to search against the UniProt database restricted to a sequence length that range < = 150 residues. A total of 1,240,895 sequences that are longer than 150 residues long were aligned with six different HMM models using the HMMSEARCH program with an E-value cutoff of 0.001. The search obtained sequences that were already known but also eight novel sequences from the phylum arthropoda and three sequences from the phylum ascomycota of the fungi kingdom.

### 4. PHI-BLAST Search of A2-like Sequences

We used PHI-BLAST 2.2.25+ to query the “nr” database (all non-redundant GenBank CDS features), using agouti related protein-2 from *S. salar* as query, filtering against false positives using the PHI pattern C-x(6)-C-x(5)-C-C-x(2)-C-x(2)-C-x-C-x(6)-C-x-C-x(6,8)-C, and reporting sequences with the pattern at position 75 and E-value WORSE than the threshold ( = 10). This is to allow for length variability in the last inter-cysteine segment, which has the length 8 in chicken, and the length 9 in human or mouse. Furthermore, we compared the 1,357 spider toxin sequences found in the “Protein” database (NCBI), with Atlantic cod *ASIP2* (and torafugu *ASIP2*).

### 5. Phylogenetic Analysis of *A1*, *A2*, and Agouti-like Sequences

A multiple sequence alignment was generated for the final set of *AgRP* and *ASIP* like sequences using MAFFT version 6 with the E-INS_I version having default parameters. The alignments were inspected and edited using Jalview (v. 2.6.1). The phylogenetic analysis was performed using a Bayesian approach as implemented in MrBayes version 3.1.2. Markov Chain Monte Carlo (MCMC) analysis was used to approximate the posterior probabilities of the trees. Analysis was run using a gamma shaped model for the variation of evolutionary rates across sites (rates = gamma) and the mixed option (aamodelpr = mixed) was used to estimate the best amino acid substitution model. Each analysis was set to run for 3,000,000 generations and every hundredth tree was sampled. A stop rule was applied to determine when to terminate the MCMC generations as recommended in the MrBayes manual (standard deviation of split frequencies <0.01). If the MCMC analysis does not hit the stop value within the default number of generations, additional generations were run for it to reach the minimum split frequencies. The first 25% of the sampled trees were discarded (burnin = 0.25) to reassure a good sample from the posterior probability distribution. A consensus tree was built from the remaining 75% of the sampled trees with the MrBayes *sumt* command using the 50% majority rule method. The *sump* command was used to control so that an adequate sample of the posterior probability distribution was reached during the MCMC procedure. The phylogenetic tree was drawn in FigTree 1.3.1 (http://tree.bio.ed.ac.uk/software/figtree/).

To root the tree, consensus sequences from arthropods used in the phylogenetic analysis were generated using HMMEMIT from HMMER3 package. First, the sequences that belong to the arthropod sequences identified in UniProt search and the spider sequences were aligned separately and separate HMM profiles were built from those alignments. Each HMM profiles serves as an input for the HMMEMIT program and a consensus sequence. were obtained using option “−C” as implemented in the HMMER3 package. The consensus sequence is formed using a plurality rule that selects the maximum probability residue at each match state from the HMM profiles.

### 6. Structure Modeling of “*A2*” Sequences and Multidimensional Scaling of RMSD Results

The three-dimensional structure of cysteine inhibitor knots (receptor binding domain) of 22 sequences was modeled using HHpred, http://toolkit.tuebingen.mpg.de/hhpred (Release-2.14.0), and MODELLER 9v3, 2008/02/01, r5971 [32]. The sequences were: Cmi *AgRP* (40 residues, ending in cys); Cmi *ASIP* (40 residues, ending in cys); Dre *AgRP2* (39 residues, ending in cys); Dre *AgRP1* (40 residues, ending in cys); Dre *ASIP1* (40 residues, ending in cys); Ola *AgRP2* (39 residues, ending in cys); Ola *AgRP1* (40 residues, ending in cys); Ola *ASIP2* (39 residues, ending in cys); Tru *AgRP1* (40 residues, ending in cys); Tru *AgRP2* (39 residues, ending in cys); Tru *ASIP1* (40 residues, ending in cys); Tru *ASIP2* (39 residues, ending in his); Tni *AgRP2* (39 residues, ending in cys); Tni *AgRP1* (40 residues, ending in cys); Tni *ASIP2* (39 residues, ending in his); Tni *ASIP1* (40 residues, ending in cys); Gac *AgRP1* (40 residues, ending in cys); Gac *AgRP2* (39 residues, ending in cys); Gac *ASIP1* (40 residues, ending in cys); Gac *ASIP2* (37 residues, ending in ala); Dla *AgRP2* (39 residues, ending in cys); Mojave Desert spider (*P. tristis*; “Ptr”) venom peptide “Plt-VI” (41 residues, ending in cys).

HHpred was used with the realign with MAC option, max. 3 HHbit iterations, scoring secondary structure, using local alignment mode, and searching against: PDB 70 18 June 2011. MODELLER 9v3, 2008/02/01, r5971, was used with default settings, manual template selection, selecting either ASIP (1y7j) (ASIP) or AgRP (1hyk) for A1 sequences and using the best template for A2 sequences, generating 22 PDB files. Pairwise global root-mean-square deviation (RMSD), based on α-carbons in all pairs of superposed structures, was obtained from SuperPose version 1 (http://wishart.biology.ualberta.ca/SuperPose/) using default settings [33]. The MatchMaker function in UCSF Chimera 1.5.3rc (http://www.cgl.ucsf.edu/chimera/docs/credits.html), an extensible molecular modeling system, was used to create a portable network image of Ptr Plt-VI (41 residues, ending in cys), using its closest neighbor in terms of RMSD distance, as a reference for superposition. Non-metric multidimensional scaling was performed using the ALSCAL algorithm [34], as implemented in SPSS Statistics 17.0, with the s-stress convergence parameter set to 0.001, and min s-stress  = >0.005. RMSD values were treated as a measure of dissimilarity. We used a square symmetric data shape; after 6 iterations, s-stress improvement was less than the threshold. A Perl script was used to convert the MDS coordinates to support vector graphics.

### 7. Use of a Sinusoidal Hough Transform to Search for Linear Synteny Between Human Chromosome 8, Region 60–100 Mb, and Medaka Chromosomes 17 or 20

Data was obtained from BioMart (http://www.biomart.org), using the ENSEMBL Genes Sanger 63 (Sanger UK) datasource, selecting as organism either *H. sapiens* (Hsa GRCh37.p3) or *O. latipes* (Ola HdrR). For human, only chromosome 8, region 60–100 Mb is selected. For medaka, chromosomes 17 and 20 are selected. From each organism, the following BioMart data fields are selected: chromosome name, gene start (bp), and “associated gene name”.

A Perl script is used to parse these data, simplifying the “associated gene name” to the first word, and excluding certain classes of genes that are likely to have ambiguous names (the source code is available upon request). Then, we define orthologues as genes that have the same name between Hsa 8 and Ola 17 or Ola 20. We create two scatterplots diagrams, one for orthologues between Hsa 8, region 60–100 Mb, and Ola 17 or Ola 20, respectively. In the scatterplots, the x and y coordinates of each point represents the gene start location in human and medaka.

Each point in the scatterplots can be transformed into a sinusoidal curve in a new system of polar coordinates (θ, ρ), where θ represents an angle and ρ represents a radius from the origin, using Duda and Hart’s version of the Hough transform [18]. The corresponding formula is (eq. 1):
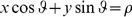
(1)


The transformation has the property that any collection of collinear points in the scatterplot will be transformed into sinusoidal curves that intersect at a common point in the polar coordinate space. Near collinearities in the scatterplot can be detected by finding regions in the polar coordinate space through which many sinusoidal curves pass.

We employ a simple sliding window approach to detect such regions. We divide the range of angles θ ∈ (0,180) into 180 bins of width 1 degree, and identify each bin with the angle at the midpoint of the range it spans. Since the values of the radius ρ are roughly of the same order of magnitude as the original gene start locations x and y, we divide the ρ dimension into bins of width 100,000. Given that 100,000 base pairs is a reasonable distance between a pair of genes in a linear synteny block, it is used here as our default setting for this parameter.

Given the sizes of the chromosomal regions being compared, we have found empirically that a range from ρ ∈ (−40 Mb, +100 Mb) is sufficient to cover the values of ρ at which sinusoidal curves intersect. We will divide this range into 1400 bins of width 0.1 Mb, and identify each bin with its midpoint value of ρ. We partition the transform space into cells, where (θ, ρ) ∈ (0,180) × (−40,100) into cells of the form C_i,j_ = (i,i+1) × (−40+0.1j, −39.9+0.1j), for all 0≤ i <180 and 0≤ j <1400. Each cell C_i,j_ corresponds to a potential collinearity along the line: x cos θ_i_ + y sin θ_i_ = ρ_j_, where θ_ I_ = i+0.5 and ρ_j_ = −39.95+0.1j.

In order to determine collinearities within the original scatterplot diagram, for each sinusoidal curve we identify those cells that are intersected by the curve and increment a counter for each of these cells. All combinations of all cells and all sinusoidal curves are evaluated leading to final intersection count of O_i,j_ for each cell C_i,j_.

Given the large evolutionary distance between human and medaka (divergence time  = 454.9 Mya), and the relatively small region considered on the human chromosome (40 Mb), it is presumed that in many cases, the largest amount of linear synteny (denoted “S”) will give a clear indication of the total amount of linear synteny in the regions being compared. While the count of orthologues in the largest linear synteny block returned by our script would mask a potential second best area of linear synteny, it would clearly recognize the difference between a case where there is no linear synteny and a case where there is some (or a large amount of) linear synteny. Another caveat is that it does not analyze the degree of clustering along the line that goes through the cluster, but given the small angle increments and the limited region considered in human, the problem of detecting linearly placed but not closely clustered points appears very limited. Thus, we have defined a simple procedure to diagnose scatterplots showing locations of orthologues in organisms, that uses as few parameters as possible.

We apply the above method to compare human chromosome 8, region 60–100 Mb, and medaka chromosomes 17 or 20. As a comparison, we also compare human chromosome 8, region 60–100 Mb, with medaka chromosomes 11 and 16 in the same way (data is obtained and preprocessed as described for medaka chromosome 17 or 20). For each comparison, we also report the number of orthologues.

### 8. Evaluation of the Background Frequency of Randomly Placed 40 Mb-sized Windows from the Human Genome that Contain More Synteny with Medaka Chromosome 17 or 20, than Human Chromosome 8, Region 60–100 Mb

Data was obtained from BioMart (http://www.biomart.org), using the ENSEMBL Genes Sanger 63 (Sanger UK) datasource, selecting as organism either *H. sapiens* (Hsa GRCh37.p3) or *O. latipes* (Ola HdrR). For human, all chromosomes are selected. For medaka, chromosomes 17 and 20 are selected. Data is obtained and preprocessed as described above.

Genomic windows of size 40 Mb are randomly placed on the human genome, using a Perl script. The windows are not allowed to overlap with human chromosome 8, region 60–100 Mb, or to overshoot chromosome ends. Each window is characterized as a positive hit to either chromosome 17 or 20 in medaka, if the number of orthologues in the region exceeded the amount of synteny observed in with human chromosome 8, region 60–100 Mb.

After sampling (N) windows, we can calculate a frequency (f) of observing “positive” windows for either chromosome 17 or 20 in medaka. We can calculate a 95% confidence interval that depends on the sample size, resting on the normal approximation of the binomial distribution, using the standard formula (eq. 2):
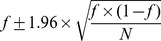
(2)


Given the number (24) and size range (50–250 Mb) of human chromosomes, 100–250 randomly placed windows of size 40 Mb would appear to give an excellent sampling of the genome. However, we continue the sampling process and follow the behaviour of the 95% confidence interval until it stabilizes, at which point we terminate the process. The sampling process is visualized using GraphPad Prism 5. The use of the normal distribution assumes that the proportions of positive and negative windows are not exceedingly close to zero.

### 9. A Control Experiment to Test Degree of Clustering on Medaka Chromosomes 17 and 20, of Orthologues Located in the Region Hsa 8, 60–100 Mb

To further investigate the 2-dimensional clustering of orthologues in the ancestral area on Hsa 8, we attempted to reverse the source and outgroup genomes in synteny database dotplots. The goal was to test if the observed clustering would be visible.

We generated a dotplot using “synteny DB dotplots” (http://teleost.cs.uoregon.edu/dotplots/), setting the source genome to Ola and the outgroup to Hsa, using the following settings: Ens61; X axis: Source; Y axis: Outgroup; Image type: Dotplot; Scale to chromosome length: no; Highlight gene of interest: no; X-axis chromosome: 17 or 20.

### 10. A Control Experiment to Evaluate the Synteny Dotplot Set of Braasch *et al*

We took the first panel of Braasch *et al*. and switched the query region to *AgRP*, instead of *ASIP*. This is because since Braasch *et al*. noted that there was no similarity between the *AgRP2* region in zebrafish and the *AgRP* region in human. However, we wanted to evaluate whether there was similarity between the *AgRP2* region in zebrafish and the ASIP region in human. Furthermore, we scanned the human genome for randomly placed 40 Mb-sized windows that superseded the ancestral Hsa 8 (60–100 Mb) area presented by Braasch *et al*. in one of the following ways: 1) Exceeding the amount of synteny with both Ola 17 and Ola 20 simultaneously, 2) Exceeding all windows in the human genome for synteny with Ola 17, 3) Exceeding all windows in human genome for linear synteny with Ola 20 (evaluated using Hough transform). Finally, we re-evaluated the similarity of the ancestral Hsa 8 (60–100 Mb) area presented by Braasch *et al*., not comparing it with *ASIP* (to which Braasch *et al*. noted similarity), but to the *AgRP* region in human. We generated dotplots (Figure 5, panels A–C) using “synteny DB dotplots” (http://teleost.cs.uoregon.edu/dotplots/), setting the source genome to Dre/Ola/Hsa, and the outgroup to Hsa, using the following settings: Ens61; X-axis: outgroup; Y-axis: source; Image type: Dotplot; Scale to chromosome length: no; Highlight gene of interest: no (but in panel B, using: *GPR158*, *ABI1*, *YME1L1*, *RAB18*, *WAC*); X-axis chromosome: Hsa 20 (30–50 Mb)/Hsa 10 (3–43 Mb)/Hsa 16 (55–75 Mb).

### 11. Search for A2-like Sequences in Little Skate, Spotted Gar, and European Eel

The little skate genome (http://skatebase.org/), *Leucoraja erinacea*, has recently become available. Little skate is a cartilagious fish that belongs to the Elasmobranchs (as opposed to the Holocephali, which include elephant shark, the genome currently considered to contain the most ancient copy of AgRP and ASIP). The spotted gar (*Lepisosteus oculatus*) and European eel (http://www.eelgenome.com/), *Anguilla anguilla*, genomes represent the last and first sequenced genomes to diverge before and after 3R whole genome duplication. We obtained query sequences of AgRP1, AgRP2, ASIP1, and ASIP2 from Fugu to search for Agouti-like sequences, using TBLASTN, as well as full-length ASIP from elephant shark.

## Supporting Information

Figure S1 **Schematic presentation of the exon-intron architecture of Agouti genes.** Boxes represent exons and connecting lines represent introns (representation does not correspond to their lengths). The forward slash between the nucleotide bases represent the intron positions. The gene structure that is available for the Agouti-like sequences in the arthropods was shown. B0W1P2 is from *C. quinquefasciatus* and A0NF98 is from *A. gambiae*.
(TIFF)

Figure S2 **Overview of orthologues located on Ola 17 and Ola 20.** The graph shows the gene start coordinates of same-name orthologues between medaka chromosome 17 (ancestral gnathostome chromosome element 1a, 1c, 19a, 19c, 10, 3b, 7b, 7c) and 20 (ancestral gnathostome chromosome element 10, 3b, 7b, 7c). The location of *AgRP2* and *ASIP2* is indicated with red bars. In the 10, 3b, 7b, and 7c region, 3–4 blocks of linear synteny can be seen, including e.g. *EMILIN1*, which is surrounded by same-name orthologues in a genomic window centered on this gene on both Ola 17 and Ola 20 (data not shown). However, only few of these genes (*NCOA2*, *TRIM55*, *ARMC1*, *IMPA1*, *CRISPLD1*, and *RALYL*) are found on the Hsa 8 (60–100 Mb) region. Although these results do not entirely rule out the possibility of teleost-specific genome duplication (TSGD) of our genes of interest, *AgRP2* and *ASIP2*, they are clearly not located in a linear synteny block.
(TIFF)

Table S1 **Orthologue counts between A1- and A2-containing teleost chromosomes.**
(DOCX)

Table S2 **Sea lamprey contigs sharing orthologues with A1 or A2-containing teleost chromosomes.**
(DOCX)

Table S3 1) The comment field is accession number to previously existing related entries, such as machine annotated entries that could be replaced by our TPA entries or constitute genomic mappings of expressed sequence tags. 2) Included in Figure 1. 3) Included in Figure 2.
(DOCX)
