# Supplementary figures and images for: Identification of Distant Agouti-Like Sequences and Re-Evaluation of the Evolutionary History of the Agouti-Related Peptide (AgRP)
Source: PLoS One. 2012 Jul 16;7(7):e40982. doi: 10.1371/journal.pone.0040982 (PMC3397983; doi:10.1371/journal.pone.0040982)

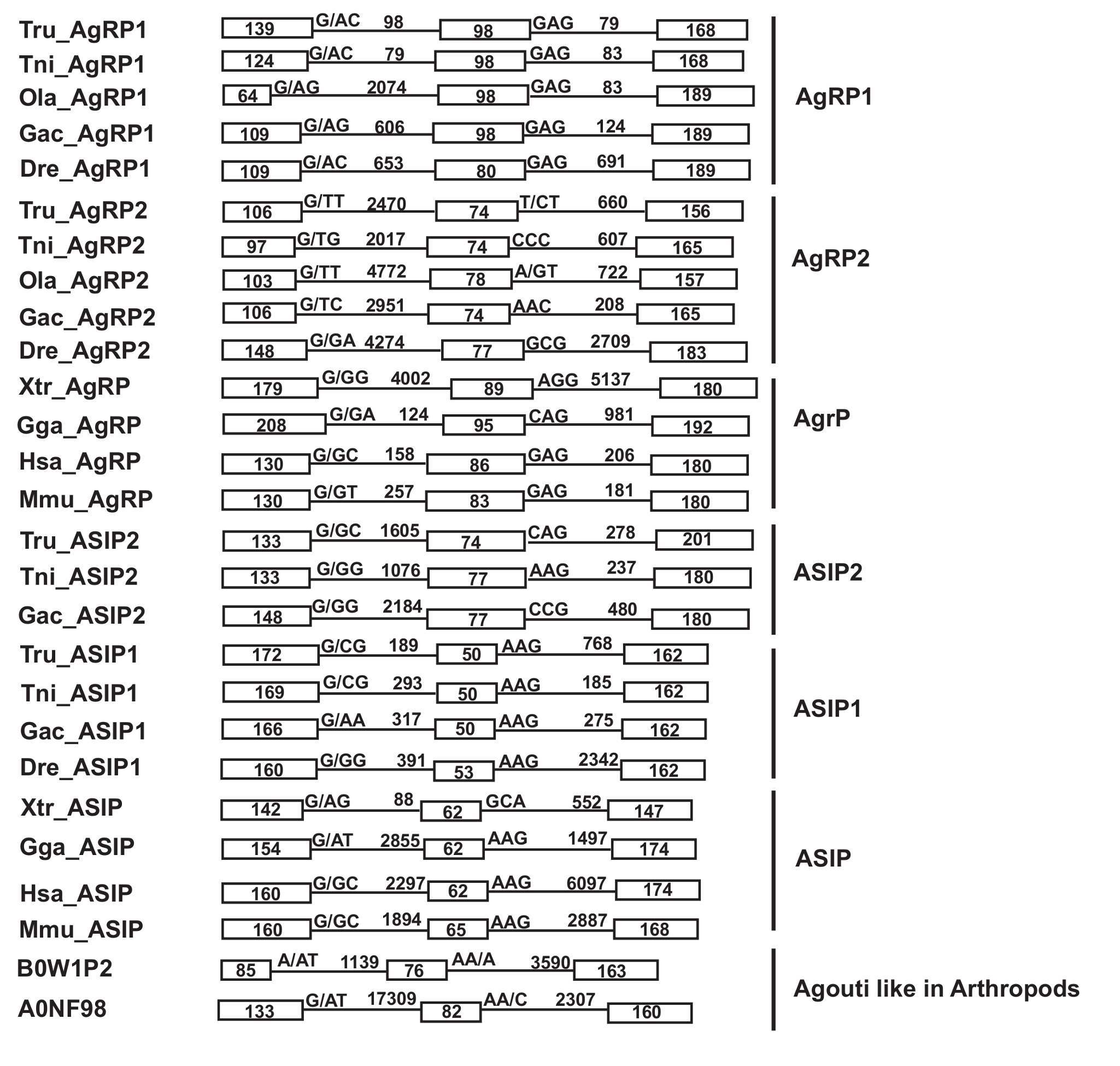

Supplement: Figure S1 — Schematic presentation of the exon-intron architecture of Agouti genes. Boxes represent exons and connecting lines represent introns (representation does not correspond to their lengths). The forward slash between the nucleotide bases represent the intron positions. The gene structure that is available for the Agouti-like sequences in the arthropods was shown. B0W1P2 is from C. quinquefasciatus and A0NF98 is from A. gambiae. (TIFF) [file pone.0040982.s001.tif]

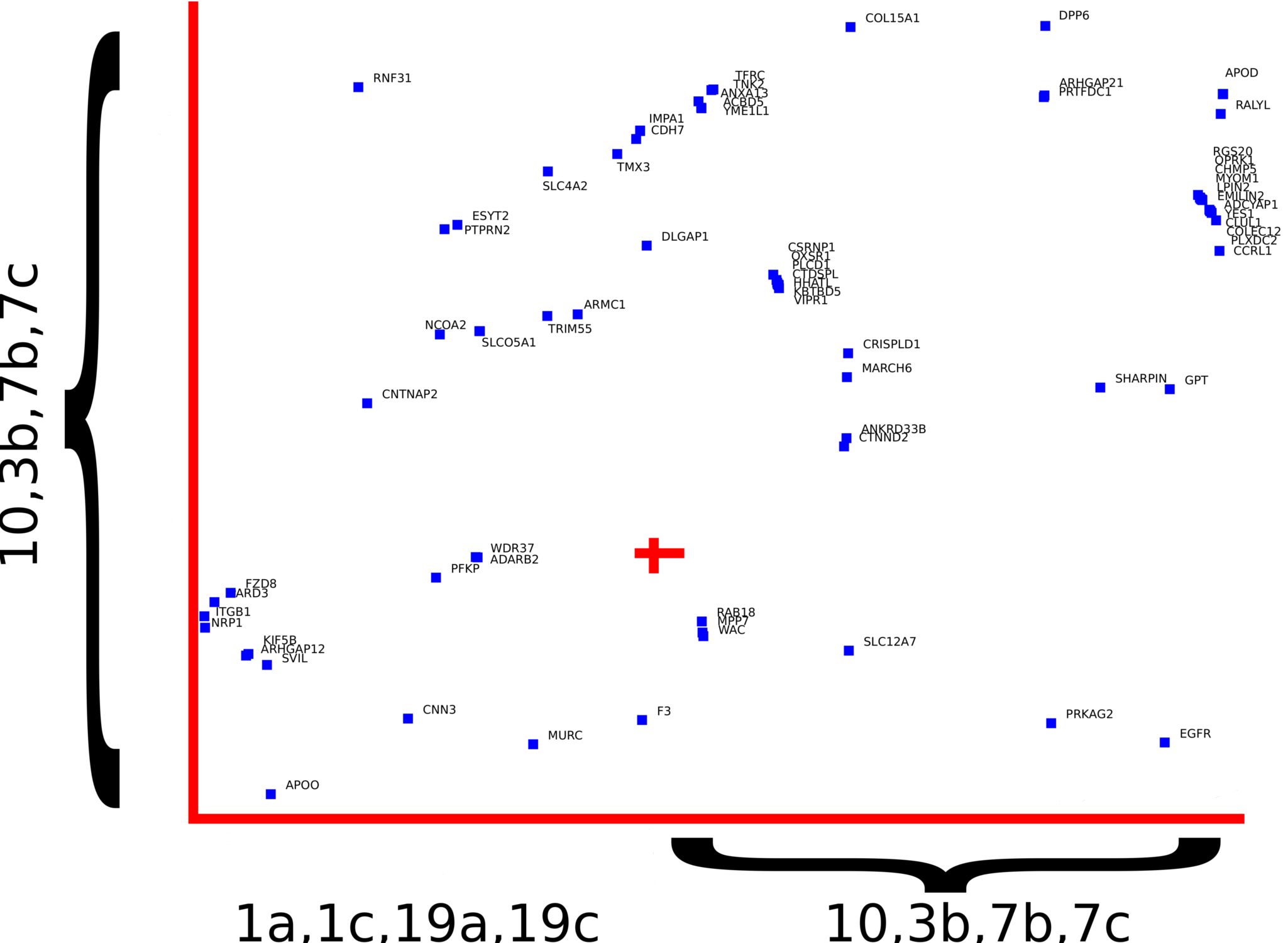

Supplement: Figure S2 — Overview of orthologues located on Ola 17 and Ola 20. The graph shows the gene start coordinates of same-name orthologues between medaka chromosome 17 (ancestral gnathostome chromosome element 1a, 1c, 19a, 19c, 10, 3b, 7b, 7c) and 20 (ancestral gnathostome chromosome element 10, 3b, 7b, 7c). The location of AgRP2 and ASIP2 is indicated with red bars. In the 10, 3b, 7b, and 7c region, 3–4 blocks of linear synteny can be seen, including e.g. EMILIN1, which is surrounded by same-name orthologues in a genomic window centered on this gene on both Ola 17 and Ola 20 (data not shown). However, only few of these genes (NCOA2, TRIM55, ARMC1, IMPA1, CRISPLD1, and RALYL) are found on the Hsa 8 (60–100 Mb) region. Although these results do not entirely rule out the possibility of teleost-specific genome duplication (TSGD) of our genes of interest, AgRP2 and ASIP2, they are clearly not located in a linear synteny block. (TIFF) [file pone.0040982.s002.tif]
