# Supplementary material for: Identification of Distant Agouti-Like Sequences and Re-Evaluation of the Evolutionary History of the Agouti-Related Peptide (AgRP)
Source: PLoS One. 2012 Jul 16;7(7):e40982. doi: 10.1371/journal.pone.0040982 (PMC3397983; doi:10.1371/journal.pone.0040982)
Supplement: Table S1 — Orthologue counts between A1- and A2-containing teleost chromosomes. (DOCX) [file pone.0040982.s003.docx]

**Supplementary Table 1: Orthologue counts between A1- and A2-containing teleost chromosomes**

|  | Dre7  (AgRP1) | Dre6  (ASIP1) | Dre2  (AgRP2) | Gac3  (AgRP2) | Gac21  (ASIP2) | Gac17  (ASIP1) | Gac2  (AgRP1) | Ola17  (AgRP2) | Ola20  (ASIP2) | Ola3  (AgRP1) | Tni5  (AgRP1) | Tni15  (AgRP2) |
| --- | --- | --- | --- | --- | --- | --- | --- | --- | --- | --- | --- | --- |
| *Dre7*  *(AgRP1)* |  |  |  |  |  |  |  |  |  |  |  |  |
| *Dre6*  *(ASIP1)* | 0 |  |  |  |  |  |  |  |  |  |  |  |
| *Dre2*  *(AgRP2)* | 11 | 8 |  |  |  |  |  |  |  |  |  |  |
| *Gac3*  *(AgRP2)* | 14 | 16 | 189 |  |  |  |  |  |  |  |  |  |
| *Gac21*  *(ASIP2)* | 23 | 0 | 32^(*)^ | 57^(*)^ |  |  |  |  |  |  |  |  |
| *Gac17*  *(ASIP1)* | 0 | 109 | 1 | 0 | 0 |  |  |  |  |  |  |  |
| *Gac2*  *(AgRP1)* | 126 | 8 | 2 | 1 | 0 | 0 |  |  |  |  |  |  |
| *Ola17*  *(AgRP2)* | 15 | 20 | 195 | 548 | 63^(*)^ | 0 | 1 |  |  |  |  |  |
| *Ola20*  *(ASIP2)* | 22 | 0 | 51^(*)^ | 83^(*)^ | 304 | 1 | 0 | 80^(*)^ |  |  |  |  |
| *Ola3*  *(AgRP1)* | 98 | 7 | 2 | 1 | 0 | 2 | 528 | 1 | 0 |  |  |  |
| *Tni5*  *(AgRP1)* | 78 | 6 | 1 | 1 | 0 | 2 | 474 | 1 | 0 | 411 |  |  |
| *Tni15*  *(AgRP2)* | 4 | 15 | 101 | 287 | 28^(*)^ | 0 | 1 | 288 | 36^(*)^ | 1 | 1 |  |

Data was obtained from BioMart (<http://www.biomart.org>), as described in Methods 4.7. The counts in the table indicate how many genes are orthologues between the chromosomes, using the sequence selection of Braasch *et al*. The highest results are clearly between “same gene” comparisons, but inter-species comparisons of chromosomes containing two different Agouti 2 genes (i.e. comparisons between the Agouti 2 gene-containing chromosomes; marked with asterisks in the table), contain more orthologue pairs than comparisons between two different A1 genes.
