# Supplementary material for: Identification of Distant Agouti-Like Sequences and Re-Evaluation of the Evolutionary History of the Agouti-Related Peptide (AgRP)
Source: PLoS One. 2012 Jul 16;7(7):e40982. doi: 10.1371/journal.pone.0040982 (PMC3397983; doi:10.1371/journal.pone.0040982)
Supplement: Table S2 — Sea lamprey contigs sharing orthologues with A1 or A2-containing teleost chromosomes. (DOCX) [file pone.0040982.s004.docx]

**Supplementary Table 2: Sea lamprey contigs sharing orthologues with A1 or A2-containing teleost chromosomes**

| ***Lamprey contig*** | ***Dre7***  ***(AgRP1)*** | ***Gac2***  ***(AgRP1)*** | ***Ola3***  ***(AgRP1)*** | ***Tni5***  ***(AgRP1)*** | ***Ola17***  ***(AgRP2)*** | ***Dre2***  ***(AgRP2)*** | ***Gac3***  ***(AgRP2)*** | ***Tni15***  ***(AgRP2)*** | ***Gac21***  ***(ASIP2)*** | ***Ola20***  ***(ASIP2)*** | ***Dre6***  ***(ASIP1)*** | ***Gac17***  ***(ASIP1)*** |
| --- | --- | --- | --- | --- | --- | --- | --- | --- | --- | --- | --- | --- |
| GL476328 |  |  | 1 | 2 | 1 |  | 1 | 1 |  |  |  |  |
| GL476330 |  | 2 | 2 | 2 | 1 |  | 1 |  | 1 | 1 |  |  |
| GL476331 |  |  |  |  |  |  |  |  |  |  | 1 | 2 |
| GL476333 | 3 | 2 | 2 | 1 | 1 |  | 1 |  |  |  |  |  |
| GL476334 |  |  |  |  | 1 |  | 2 | 1 | 1 | 2 |  |  |
| GL476335 |  |  |  |  |  |  | 1 |  |  |  | 3 | 4 |
| GL476336 |  |  |  |  | 2 |  | 2 | 1 |  |  | 1 |  |
| GL476340 |  |  |  |  |  |  |  |  |  |  |  | 2 |
| GL476341 |  | 1 |  |  | 2 | 1 | 2 |  | 1 | 1 |  |  |
| GL476346 | 1 | 2 | 2 | 1 | 1 |  | 1 | 1 | 1 | 1 |  |  |
| GL476349 |  |  |  |  | 2 |  |  | 2 | 2 |  |  |  |
| GL476350 |  |  |  |  |  |  |  |  | 1 | 1 | 1 | 4 |
| GL476354 | 2 | 3 | 2 | 2 |  |  | 1 |  | 1 | 1 |  |  |
| GL476355 |  | 1 | 1 | 1 | 1 |  | 2 | 1 |  |  |  | 1 |
| GL476358 |  | 4 | 2 | 2 | 2 |  | 3 | 1 |  |  | 2 |  |
| GL476370 |  | 1 |  | 1 | 1 |  | 1 |  |  |  | 2 |  |
| GL476373 |  |  |  |  |  |  |  |  |  |  | 4 |  |
| GL476376 |  |  |  |  | 2 |  | 2 | 2 |  |  |  |  |
| GL476395 | 1 | 3 | 3 | 3 |  |  |  |  |  |  |  |  |
| GL476396 |  |  |  |  | 1 |  | 1 | 1 |  |  |  | 2 |
| GL476398 |  |  |  |  |  |  |  |  |  |  | 4 |  |
| GL476399 | 1 | 7 | 6 | 1 | 2 |  | 2 | 1 |  |  |  |  |
| GL476414 | 2 |  |  |  | 1 | 1 | 1 |  |  |  |  |  |
| GL476415 |  | 1 | 1 | 1 |  |  | 1 |  |  |  |  | 2 |
| GL476421 |  |  |  |  | 2 |  | 2 |  |  |  |  |  |
| GL476426 |  | 1 | 1 | 1 | 2 |  | 3 | 2 |  |  | 1 |  |
| GL476434 | 1 | 2 | 2 | 2 |  |  |  |  |  |  | 1 |  |
| GL476446 | 1 | 1 | 1 | 1 | 2 | 2 | 3 | 3 |  |  |  |  |
| GL476458 |  | 1 | 1 | 1 |  |  |  |  |  |  | 2 |  |
| GL476459 | 2 | 2 | 2 |  |  |  |  |  |  |  |  |  |
| GL476466 |  |  |  |  | 2 |  |  | 2 | 3 | 3 |  |  |
| GL476473 |  |  |  |  |  |  |  |  |  |  |  | 6 |
| GL476493 |  |  |  |  |  |  |  |  |  |  |  | 3 |
| GL476508 |  | 2 | 2 | 2 |  |  |  |  |  |  |  |  |
| GL476522 |  |  |  |  | 2 | 1 | 2 | 1 | 1 | 1 |  |  |
| GL476524 |  |  |  |  |  | 4 | 5 | 4 |  |  |  |  |
| GL476531 |  | 2 | 2 | 2 |  |  |  |  |  |  |  |  |
| GL476533 |  |  |  |  |  |  |  |  |  |  |  | 2 |
| GL476535 |  | 2 | 2 | 1 |  |  |  |  |  |  |  | 1 |
| GL476538 | 5 | 1 | 1 | 1 |  |  |  |  |  |  |  |  |
| GL476540 | 1 |  | 1 | 2 |  |  |  |  |  |  |  |  |
| GL476542 |  |  |  |  |  |  |  |  |  |  | 1 | 2 |
| GL476553 |  |  |  |  |  |  |  |  |  |  |  | 2 |
| GL476561 |  |  |  |  |  |  |  |  | 1 | 2 |  |  |
| GL476566 |  | 3 | 4 | 2 |  |  |  |  |  |  |  |  |
| GL476569 |  |  |  |  |  |  |  |  |  |  |  | 2 |
| GL476573 |  |  |  |  |  |  |  |  | 2 | 2 |  |  |
| GL476583 |  | 2 |  |  |  |  |  |  |  |  |  |  |
| GL476588 | 1 | 3 | 3 | 3 |  |  |  |  |  |  |  |  |
| GL476589 |  |  |  |  | 2 |  |  | 2 |  |  |  |  |
| GL476590 | 5 | 5 |  | 4 |  |  |  |  | 3 | 3 | 1 |  |
| GL476598 |  | 2 | 2 | 2 |  |  |  |  |  |  |  |  |
| GL476601 |  | 8 | 4 |  |  |  |  |  |  |  |  |  |
| GL476610 |  |  |  |  |  |  |  |  |  |  | 2 | 2 |
| GL476616 | 1 | 3 | 3 | 1 |  | 1 |  |  | 1 | 2 |  | 1 |
| GL476617 |  | 4 | 4 |  |  |  |  |  |  |  |  |  |
| GL476636 |  |  |  |  |  |  |  |  |  |  | 1 | 2 |
| GL476650 | 3 | 3 | 3 | 1 | 1 |  | 1 | 1 |  |  |  |  |
| GL476652 |  |  |  |  | 3 |  | 3 | 3 | 1 | 3 |  |  |
| GL476656 |  |  |  |  | 2 |  | 2 |  |  |  |  |  |
| GL476657 |  |  |  |  |  |  |  |  |  |  |  | 2 |
| GL476661 |  |  |  |  | 2 |  |  |  |  |  |  |  |
| GL476673 |  |  |  |  |  |  |  |  |  |  |  | 4 |
| GL476675 |  | 2 |  | 2 |  |  |  |  |  |  |  |  |
| GL476697 |  |  |  |  |  |  | 6 |  | 1 | 1 |  |  |
| GL476698 |  | 2 | 2 | 2 |  |  |  |  |  |  |  |  |
| GL476705 |  | 2 | 2 | 2 |  |  |  |  |  |  |  |  |
| GL476732 |  | 6 | 5 | 7 |  |  |  |  |  |  |  |  |
| GL476736 | 1 |  |  |  | 3 | 1 | 3 | 1 | 3 | 4 |  |  |
| GL476764 |  |  |  |  | 1 |  | 1 | 1 | 2 | 2 |  |  |
| GL476768 | 2 |  |  |  |  |  |  |  |  |  |  |  |
| GL476773 |  |  |  |  | 6 |  | 6 |  |  |  |  |  |
| GL476775 |  |  |  |  |  |  |  |  |  |  |  | 2 |
| GL476790 |  |  |  |  | 1 | 1 | 1 |  | 2 | 2 |  |  |
| GL476793 |  | 2 | 1 | 2 |  |  |  |  |  |  |  |  |
| GL476846 |  |  |  |  | 1 | 1 |  |  | 3 | 3 |  |  |
| GL476874 | 1 |  |  |  | 3 | 1 | 4 |  | 2 | 2 |  |  |
| GL476919 |  | 2 | 2 | 1 |  |  |  |  |  |  |  |  |
| GL476931 | 1 | 2 |  | 1 |  |  |  |  |  |  |  |  |
| GL476932 | 1 | 1 | 2 | 1 |  |  |  |  |  |  |  |  |
| GL476937 |  |  |  |  |  |  | 3 |  |  |  |  |  |
| GL476950 |  |  |  |  | 2 | 1 | 2 | 1 | 1 | 1 |  | 1 |
| GL476967 | 1 |  |  |  |  |  |  |  | 2 | 2 |  | 1 |
| GL476989 | 2 | 1 | 1 | 1 |  |  |  |  |  |  |  |  |
| GL477001 |  |  |  |  | 5 | 1 | 5 |  | 1 | 1 |  |  |
| GL477010 |  |  |  |  | 2 |  |  |  |  |  |  |  |
| GL477029 | 1 | 1 | 1 | 1 | 1 |  | 1 | 2 |  |  |  |  |
| GL477033 | 1 |  |  |  | 1 | 1 | 2 |  | 1 |  |  | 1 |
| GL477040 |  | 1 | 1 |  |  | 1 |  |  |  |  |  | 2 |
| GL477046 |  |  |  |  |  |  |  |  |  |  |  | 2 |
| GL477069 |  |  |  |  |  |  | 1 |  |  |  |  | 2 |
| GL477076 |  | 2 |  |  |  |  |  |  |  |  |  |  |
| GL477077 |  |  |  |  | 2 |  | 2 |  | 1 | 1 |  |  |
| GL477086 |  |  |  |  |  |  |  |  |  |  |  | 2 |
| GL477101 |  |  |  |  | 1 |  | 1 | 1 | 2 | 1 |  |  |
| GL477112 | 4 | 1 | 1 | 1 |  |  |  |  |  |  |  |  |
| GL477137 |  | 1 | 3 | 3 |  |  | 1 |  |  |  |  | 1 |
| GL477159 |  |  |  |  | 2 |  | 2 |  |  |  |  |  |
| GL477191 |  |  |  |  |  |  |  |  |  |  |  | 2 |
| GL477226 |  |  |  |  |  |  | 1 | 2 |  |  |  |  |
| GL477255 |  |  |  |  | 2 |  | 2 |  |  |  |  |  |
| GL477259 |  |  |  |  |  |  |  |  |  |  |  | 2 |
| GL477274 | 2 |  |  |  |  |  |  |  |  |  |  |  |
| GL477289 |  | 1 | 1 | 2 |  |  |  |  |  |  |  |  |
| GL477323 |  | 6 | 5 | 6 |  |  |  |  |  |  |  |  |
| GL477329 | 2 | 1 | 1 | 1 |  |  |  |  |  |  |  |  |
| GL477332 |  |  |  |  | 1 | 1 | 2 |  |  |  |  |  |
| GL477336 |  | 2 | 2 | 2 |  |  |  |  |  |  |  |  |
| GL477361 |  |  |  |  |  |  |  |  | 2 | 2 |  |  |
| GL477373 |  |  |  |  |  |  |  |  | 4 | 1 |  |  |
| GL477431 |  |  |  |  |  |  |  |  | 2 | 2 |  |  |
| GL477437 |  |  |  |  |  |  |  |  |  |  |  | 2 |
| GL477450 |  |  |  |  |  |  |  |  |  |  |  | 2 |
| GL477471 |  |  |  |  |  |  |  |  |  |  |  | 2 |
| GL477547 |  |  |  |  |  |  |  |  |  |  |  | 2 |
| GL477554 | 2 | 1 |  |  |  |  |  |  |  |  |  |  |
| GL477555 |  |  |  |  | 2 |  | 2 | 2 |  | 2 |  |  |
| GL477608 |  | 2 | 2 | 2 |  |  |  |  |  |  |  |  |
| GL477625 | 1 | 1 | 1 | 1 |  |  |  |  |  |  | 3 |  |
| GL477674 | 2 | 3 | 1 | 1 |  |  |  |  |  |  |  |  |
| GL477760 |  |  |  |  |  |  |  |  |  |  | 1 | 2 |
| GL477791 |  |  |  |  | 2 |  | 2 |  |  |  |  |  |
| GL477881 |  | 2 | 1 | 2 |  |  |  |  |  |  |  | 1 |
| GL477929 |  |  |  |  | 2 |  | 1 |  |  |  |  |  |
| GL477954 |  | 2 | 2 | 2 |  |  |  |  |  |  |  |  |
| GL477996 |  |  |  |  | 2 |  |  | 2 | 2 |  |  |  |
| GL478075 |  |  |  |  |  |  |  |  | 2 | 2 |  |  |
| GL478094 |  |  |  |  | 2 |  | 2 |  |  |  |  |  |
| GL478108 |  |  |  |  | 2 |  | 1 |  |  |  |  |  |
| GL478117 |  | 2 |  |  |  |  |  |  |  |  |  |  |
| GL478165 |  | 2 | 2 |  |  |  |  |  |  |  |  |  |
| GL478211 |  |  |  |  |  |  | 2 |  |  |  |  |  |
| GL478283 |  |  |  |  | 2 |  | 2 |  |  |  |  |  |
| GL478495 |  |  |  |  |  |  |  |  |  |  |  | 2 |
| GL478791 |  |  |  |  |  |  |  |  |  |  | 2 | 1 |
| GL479196 |  |  |  |  | 2 |  |  |  |  |  |  |  |
| GL479225 |  |  |  |  |  |  | 2 |  |  |  |  |  |
| GL479284 |  |  |  |  |  |  |  |  |  |  |  | 2 |
| GL479567 | 2 |  |  |  |  |  |  |  |  |  |  |  |
| GL479714 |  |  |  |  |  |  |  |  | 2 | 2 |  |  |
| GL480173 | 2 |  |  |  |  |  |  |  |  |  |  |  |
| GL481013 |  |  |  |  | 1 |  | 1 | 2 |  |  |  |  |
| GL481660 | 2 |  |  |  |  |  |  |  |  |  |  |  |
| GL482992 | 2 |  |  |  |  |  |  |  |  |  |  |  |
| GL483536 |  |  |  |  |  | 4 | 4 | 4 |  |  |  |  |
| GL484797 | 2 |  |  |  |  |  |  |  |  |  |  |  |
| GL485814 | 2 |  |  |  |  |  |  |  |  |  |  |  |
| GL488057 |  |  |  |  | 1 |  | 1 | 2 |  |  |  |  |
| GL492181 |  | 2 | 2 | 2 |  |  |  |  |  |  |  |  |
| GL495392 |  |  |  |  |  |  |  |  |  |  |  | 2 |
| GL501070 |  |  |  |  | 2 |  | 2 | 2 |  | 2 |  |  |

Data was obtained from BioMart (<http://www.biomart.org>), and pre-processed as described in Methods 4.7. We compared the query chromosomes, selected using the gene set from Braasch *et al*., with ENSEMBL Genes 64 (Sanger UK) *P. marinus* 7.0. Contigs that have more than one hit, for any query chromosome, are listed. As in online appendix (Supplementary Table 1), there is a higher number of contigs containing paralogies with AgRP2 and ASIP2 teleots chromosomes as compared with contigs containing simultaneous paralogies with ASIP1 and AgRP1 teleost chromosomes, potentially suggesting an older common origin than TSGD (R3). There are 15 contigs that link AgRP2 and ASIP2, 7 contigs that link A1 and AgRP2, 7 contigs that link AgRP1 and AgRP2, 6 contigs that link A2 to AgRP, 5 contigs that link A1 together, 4 contigs that link AgRP2 with ASIP1, 2 contigs that link A1 to ASIP2, 1 contig that links A2 to ASIP1, and 1 contig that links ASIP2 to ASIP1. Because the genome is not assembled, we can not determine if the ancestral region containing the A2 genes in teleosts was duplicated in R1 before lamprey.
