# Supplementary material for: Identification of Distant Agouti-Like Sequences and Re-Evaluation of the Evolutionary History of the Agouti-Related Peptide (AgRP)
Source: PLoS One. 2012 Jul 16;7(7):e40982. doi: 10.1371/journal.pone.0040982 (PMC3397983; doi:10.1371/journal.pone.0040982)
Supplement: Table S3 — 1) The comment field is accession number to previously existing related entries, such as machine annotated entries that could be replaced by our TPA entries or constitute genomic mappings of expressed sequence tags. 2) Included in Figure 1. 3) Included in Figure 2. (DOCX) [file pone.0040982.s005.docx]

| Species | Name | Length | Acc. number | TPA | Previous entry^1)^ | Align.^2)^ | Cons. | Phy.^3)^ |
| --- | --- | --- | --- | --- | --- | --- | --- | --- |
| *Salmo salar* | AgRP2 | 117 | NM_001146678 |  |  | Yes |  | Yes |
| *Oryzias latipes* | AgRP2 | 114 | BR000920 | Yes | mRNA, AM300511 | Yes |  | Yes |
| *Dicentrarchus labrax* | AgRP2 | 116 | HE660087 |  | AM983668.1, FM011395.1, FM008100.1, FM018657.1 | Yes |  | Yes |
| *Gasterosteus aculeatus* | AgRP2 | 115 | BR000927 | Yes | GENSCAN00000013027 | Yes |  | Yes |
| *Gadus morhua* | AgRP2 | 43 | BR000936 | Yes |  | Yes |  | Yes |
| *Takifugu rubripes* | AgRP2 | 115 | ENSTRUP00000009355 |  |  | Yes |  | Yes |
| *Tetraodon nigroviridis* | AgRP2 | 114 | ENSTNIP00000010647 |  |  | Yes |  | Yes |
| *Danio rerio* | AgRP2 | 136 | BR000926 | Yes | mRNA, CD751231 | Yes |  | Yes |
| *Anguilla anguilla* | AgRP2-1 | 102 | Scaffold 9054 |  |  | Yes |  |  |
| *Anguilla anguilla* | AgRP2-2 | 47 | Scaffold 1167 |  |  | Yes |  |  |
| *Lepisosteus oculatus* | AgRP2 | 130 | BR000972 | Yes |  | Yes |  |  |
| *Ptyochromis sp.* | ASIP2 | 156 | BJ703322 |  |  | Yes |  | Yes |
| *Takifugu rubripes* | ASIP2 | 136 | ENSTRUP00000005680 |  |  | Yes |  | Yes |
| *Tetraodon nigroviridis* | ASIP2 | 130 | BR000929 | Yes |  | Yes |  | Yes |
| *Gasterosteus aculeatus* | ASIP2 | 135 | BR000928 | Yes | GENSCAN00000027758 | Yes |  | Yes |
| *Gadus morhua* | ASIP2 | 136 | BR000935 | Yes |  | Yes |  | Yes |
| *Oryzias latipes* | ASIP2 | 149 | BR000919 | Yes | mRNA, BJ487471 | Yes |  | Yes |
| *Oncorhynchus mykiss* | AgRP1 | 81 | CR376289 |  |  | Yes |  | Yes |
| *Gadus morhua* | AgRP1 | 128 | BR000938 | Yes |  | Yes |  | Yes |
| *Dicentrarchus labrax* | AgRP1 | 142 | HE660086 |  |  | Yes |  | Yes |
| *Gasterosteus aculeatus* | AgRP1 | 132 | BR000932 | Yes | GENSCAN00000018994 | Yes |  | Yes |
| *Oryzias latipes* | AgRP1 | 117 | BR000923 | Yes | FM167327, AM140194 | Yes |  | Yes |
| *Takifugu rubripes* | AgRP1 | 135 | ENSTRUP00000009367 |  |  | Yes |  | Yes |
| *Tetraodon nigroviridis* | AgRP1 | 130 | ENSTNIP00000013124 |  |  | Yes |  | Yes |
| *Danio rerio* | AgRP1 | 126 | ENSDARP00000116390 |  |  | Yes |  | Yes |
| *Homo sapiens* | AgRP | 132 | ENSP00000290953 |  |  | Yes |  | Yes |
| *Canis familiaris* | AgRP | 132 | XP_853932 |  |  | Yes |  |  |
| *Bos taurus* | AgRP | 134 | CAA05148.1 |  |  | Yes |  |  |
| *Mus musculus* | AgRP | 131 | ENSMUSP00000005849 |  |  | Yes |  | Yes |
| *Equus caballus* | AgRP | 131 | XP_001497739.1 |  |  | Yes |  |  |
| *Callithrix jacchus* | AgRP | 132 | XP_002761218.1 |  |  | Yes |  |  |
| *Rattus norvegicus* | AgRP | 129 | NP_387499.1 |  |  | Yes |  |  |
| *Monodelphis domestica* | AgRP | 179 | XP_001373210.1 |  |  | Yes |  |  |
| *Gallus gallus* | AgRP | 165 | ENSGALP00000003505 |  |  | Yes |  | Yes |
| *Ornithorhynchus anatinus* | AgRP | 140 | LOC100075038 |  |  | Yes |  |  |
| *Xenopus tropicalis* | AgRP | 148 | BR000931 | Yes | XM_002937321 | Yes |  | Yes |
| *Raja erinacea* | AgRP | 48 | Contig674736 |  |  | Yes |  |  |
| *Gadus morhua* | ASIP1 | 49 | BR000937 | Yes |  | Yes |  | Yes |
| *Scophthalmus maximus* | ASIP1 | 123 | HE598752 |  |  | Yes |  | Yes |
| *Gasterosteus aculeatus* | ASIP1 | 126 | FR872817 | Yes |  | Yes |  | Yes |
| *Solea senegalensis* | ASIP1 | 132 | HE598753 |  |  | Yes |  | Yes |
| *Takifugu rubripes* | ASIP1 | 128 | ENSTRUP00000008132 |  |  | Yes |  | Yes |
| *Tetraodon nigroviridis* | ASIP1 | 127 | ENSTNIP00000003081 |  |  | Yes |  | Yes |
| *Dicentrarchus labrax* | ASIP1 | 137 | FM021895.1 |  |  | Yes |  | Yes |
| *Danio rerio* | ASIP1 | 125 | ENSDARP00000100167 |  |  | Yes |  | Yes |
| *Xenopus tropicalis* | ASIP | 117 | BR000930 | Yes |  | Yes |  | Yes |
| *Monodelphis domestica* | ASIP | 130 | ENSMODP00000003361 |  |  | Yes |  |  |
| *Callorhinchus milii* | ASIP | 141 | BR000915 | Yes | FAA00700.1, FAA00709.1, FAA00701.2 | Yes |  | Yes |
| *Homo sapiens* | ASIP | 132 | ENSP00000364092 |  |  | Yes |  | Yes |
| *Equus caballus* | ASIP | 133 | ENSECAP00000003317 |  |  | Yes |  |  |
| *Mus musculus* | ASIP | 131 | ENSMUSP00000029123 |  |  | Yes |  | Yes |
| *Rattus norvegicus* | ASIP | 131 | NP_443211 |  |  | Yes |  |  |
| *Canis familiaris* | ASIP | 131 | NP_001007264.1 |  |  | Yes |  |  |
| *Gallus gallus* | ASIP | 139 | ENSGALP00000034003 |  |  | Yes |  | Yes |
| *Raja erinacea* | ASIP | 155 | AESE011535652, AESE011594554, AESE011079059 |  |  | Yes |  |  |
| *Plectreurys tristis* | Plt-VI | 82 | AAC47207.1 |  |  | Yes | SPTR_cons |  |
| *Plectreurys tristis* | Plt-VIII | 61 | AAC47208.1 |  |  | Yes | SPTR_cons |  |
| *Plectreurys tristis* | Plt-XI | 79 | AAC47201.1 |  |  | Yes | SPTR_cons |  |
| *Haplopelma schmidti* | HWTX-XVIa4 | 116 | B3FIP8_HAPSC |  |  | Yes | Arth_cons |  |
| *Culex quinquefasciatus* | Uncharacterized protein | 107 | B0W1P2_CULQU |  |  | Yes | Arth_cons |  |
| *Anopheles gambiae* | Uncharacterized protein | 125 | A0NF98_ANOGA |  |  | Yes | Arth_cons |  |
| *Harpegnathos saltator* | Uncharacterized protein | 68 | E2B7Z7_9HYME |  |  | Yes | Arth_cons |  |
| *Camponotus floridanus* | Uncharacterized protein | 100 | E2ADU7_9HYME |  |  | Yes | Arth_cons |  |
| *Solenopsis invicta* | Uncharacterized protein | 87 | E9ISS2_SOLIN |  |  | Yes | Arth_cons |  |
| *Daphnia pulex* | Uncharacterized protein | 70 | E9FSX5_DAPPU |  |  | Yes | Arth_cons |  |
| *Drosophila willistoni* | Uncharacterized protein | 92 | B4NHX5_DROWI |  |  | Yes | Arth_cons |  |
| *Botryotinia fuckeliana* | Uncharacterized protein | 141 | A6RQC0_BOTFB |  |  | Yes | Arth_cons |  |
| *Nectria haematococca* | Uncharacterized protein | 134 | C7ZKX5_NECH7 |  |  | Yes | Arth_cons |  |
| *Chaetomium globosum* | Uncharacterized protein | 148 | Q2HBM0_CHAGB |  |  | Yes | Arth_cons |  |
| *Haplopelma hainanum* | Hainantoxin-1.3 | 83 | H1A03_HAPHA |  |  | Yes | Arth_cons |  |
| *Chilobrachys guangxiensis* | Guangxiensistoxin | 103 | JZT60_CHIJI |  |  | Yes | Arth_cons |  |
| *Oncorhynchus mykiss* | AgRP2 | 118 | CA343080 |  | CA388224 | No |  | No |
| *Sparus aurata* | AgRP2 | 116 | AM960492.1 |  |  | No |  | Yes |
| *Dissostichus mawsoni* | AgRP2 | 116 | FE193792.1 |  |  | No |  | Yes |
| *Osmerus mordax* | AgRP2 | 116 | EL530902.1 |  |  | No |  | Yes |
| *Cyprinus carpio* | AgRP2 | 135 | DW723129.1 |  |  | No |  | Yes |
| *Ictalurus punctatus* | ASIP2 | 120 | FD261964.1 |  |  | No |  | Yes |
| *Oreochromis niloticus* | ASIP2 | 156 | BR000940 | Yes | BJ703322 | No |  | Yes |
| *Oreochromis niloticus* | AgRP2 | 116 | BR000939 | Yes |  | No |  | Yes |
| *Latimeria chalumnae* | AgRP2 or ASIP2 | Unknown | AFYH01237232 |  |  | No |  | No |

**Supplementary table S3**

1. The comment field is accession number to previously existing related entries, such as machine annotated entries that could be replaced by our TPA entries or constitute genomic mappings of expressed sequence tags.
2. Included in Figure 1.
3. Included in Figure 2.
